# Implementation and translation of evidence-based dietary strategies for type 2 diabetes in resource-limited settings: a scoping review based on the CFIR framework

**DOI:** 10.3389/fpubh.2026.1870603

**Published:** 2026-08-06

**Authors:** Kaixi Shao, Haitao Lei, Junjie Zhu, Xinyi Chen, Yongzong Sina, Runlan Yao

**Affiliations:** 1 School of Nurse, Dali University, Dali, China; 2 Dali First People's Hospital, Dali, China

**Keywords:** Consolidated Framework for Implementation Research (CFIR), dietary strategies, health services research, implementation science, resource-limited settings, type 2 diabetes mellitus

## Abstract

**Introduction:**

Dietary intervention is the core of type 2 diabetes mellitus (T2DM) management, yet substantial gaps exist between evidence-based dietary guidelines and real-world implementation in resource-limited settings including low- and middle-income countries (LMICs) and marginalized subgroups within high-income nations. Implementation science frameworks such as the updated Consolidated Framework for Implementation Research (CFIR 2.0) enable systematic analysis of multi-level barriers and facilitators of dietary strategies, but comprehensive cross-regional synthesis targeting resource-scarce populations remains scarce. This scoping review aims to map, categorize, and interpret factors shaping the translation of evidence-based T2DM dietary interventions across resource-limited contexts using the CFIR 2.0 framework.

**Methods:**

This scoping review strictly followed PRISMA-ScR reporting standards. We systematically searched PubMed, Web of Science, CNKI and other Chinese/English databases from database inception to March 1, 2026 for quantitative, qualitative and mixed-method studies focused on the implementation, barriers, enablers or cultural adaptation of T2 dietary interventions in resource-limited populations. Two independent reviewers completed study screening, full-text eligibility assessment and CFIR deductive thematic coding; disagreements were resolved via group discussion. Co-occurrence network analysis visualized interconnections between implementation themes, and stratified comparative synthesis was performed to distinguish disparities between LMICs and disadvantaged subgroups in high-income countries.

**Results:**

A total of 643 initial records were retrieved, and 113 eligible studies covering Asia, Africa, North America and other regions were finally included. Five core CFIR domains jointly determine implementation outcomes, with localized cultural adaptation serving as the overarching central theme: 1. Individual characteristics: Knowledge and skill deficits (85/113 studies) and biased health attitudes were dominant barriers; peer education and targeted belief reshaping acted as key facilitators. 2. Outer setting: Food deserts, mismatched sociocultural eating norms and insufficient public health policy investment hindered adherence, while community organizations, religious stakeholder collaboration and revitalized traditional food culture boosted intervention uptake. 3. Inner setting: Shortages of nutrition professionals and incomplete primary care structural systems formed institutional barriers; standardized diabetes clinics and multi-disciplinary cooperation delivered organizational support. 4. Intervention characteristics: Generic one-size-fits-all dietary tools lacked contextual adaptability, while low-carb protocols and stage-matched personalized nutrition plans possessed robust clinical evidence and high implementability after local co-design. 5. Implementation process: Lack of standardized training for frontline staff was a major barrier; layered offline group courses, low-tech paper tracking tools and voice-based mobile reminders facilitated long-term execution. Notable disparities existed across two resource-limited population groups: LMIC implementation obstacles mainly stemmed from nationwide systemic underinvestment, whereas marginalized ethnic subgroups in high-income countries faced barriers rooted in colonial erosion of traditional food systems and racial health inequities. Co-occurrence network analysis confirmed knowledge-skill gaps as the central bridging factor linking all five CFIR domains. Evidence gaps included insufficient long-term follow-up studies (only 12% of studies tracked participants over 2 years) and limited data from African and rural Asian primary care contexts.

**Discussion:**

Sustained, effective translation of T2DM dietary interventions cannot rely on single-point improvements, but requires synergistic optimization across all five CFIR domains centered on localized cultural adaptation. LMICs and high-income vulnerable subgroups demand differentiated intervention strategies due to divergent structural root causes. Four actionable multi-level recommendations are proposed: strengthening national chronic disease policy and grassroots nutrition resource investment; systematic nutrition capacity training for primary care providers; developing context-customized, simplified visual dietary tools; and building integrated hospital-community-family long-term follow-up support systems. Limitations of this review include exclusive Chinese-English literature retrieval and absence of formal bias risk evaluation. Future research should conduct hybrid implementation-effect trials, develop scalable CFIR+RE-AIM combined toolkits and carry out long-term multi-center studies in understudied African and rural Asian regions.

**Systematic review registration:**

https://osf.io/j2zh9/.

## 1 Introduction

Type 2 diabetes mellitus (T2DM) represents a major global public health challenge, particularly in resource-limited settings such as low- and middle-income countries (LMICs) (1). Data from the International Diabetes Federation (IDF) indicate that approximately 81% of people with diabetes worldwide live in LMICs, and the disease burden is growing rapidly (1). Evidence-based dietary management is the cornerstone of T2DM prevention and control, as it can effectively improve glycemic control and even achieve disease remission (2). However, in real-world practice in LMICs, the implementation and translation of these strategies face substantial difficulties, with a persistent gap between guideline evidence and primary care practice. Implementation science offers key frameworks for bridging this gap. Among these, the Consolidated Framework for Implementation Research (CFIR) is widely used to analyze barriers and facilitators of complex interventions, with its systematic multi-level structure encompassing five core domains: intervention characteristics, outer setting, inner setting, individual characteristics, and implementation process (3, 4). While CFIR has been increasingly adopted in chronic disease management and behavioural health (5), empirical evidence remains highly fragmented for its application to T2DM dietary strategies specifically in LMICs (4).

However, systematic evidence synthesis using CFIR for T2DM dietary interventions in LMICs remains scarce, hindering the identification of common barriers and context-specific implementation mechanisms across regions and cultures. Therefore, this study conducted a scoping review using the CFIR framework to synthesize evidence on the implementation of evidence-based dietary strategies for T2DM in LMICs. Specifically, we aimed to identify core barriers and facilitators, clarify cross-domain interactions, and provide an integrated analytical framework to inform the design, adaptation, and evaluation of culturally feasible dietary interventions, ultimately improving the quality of primary diabetes care in resource-limited settings.

## 2 Materials and methods

### 2.1 Study design and protocol registration

This study is a scoping review designed and reported in strict accordance with the Preferred Reporting Items for Systematic Reviews and Meta-Analyses Extension for Scoping Reviews (PRISMA-ScR) guidelines. The study protocol was pre-registered on the Open Science Framework (OSF) prior to study initiation.

### 2.2 Research questions

Based on the Consolidated Framework for Implementation Research (CFIR), this study proposes the following specific research questions: (1) Mapping: What are the distribution characteristics of existing studies in terms of geographic regions, target populations, intervention types, and study designs? (2) Determinants: Among the five CFIR domains (intervention characteristics, outer setting, inner setting, individual characteristics, and implementation process), which factors are reported as major barriers or facilitators to implementation?

### 2.3 Inclusion and exclusion criteria

The scope of the study was defined using the PCC framework (Participants, Concept, Context), with operationalized decision rules developed.

#### 2.3.1 Inclusion criteria

(1) Population: Patients diagnosed with type 2 diabetes mellitus (T2DM) in resource-limited settings (including various subgroups), as well as primary healthcare providers, managers, or community workers involved in the implementation of dietary interventions for T2DM in these settings. (2) Concept: Any evidence-based dietary/nutritional intervention for T2DM, with studies addressing at least one of the following: implementation process, barriers/facilitators, cultural adaptation, or implementation outcome evaluation. (3) Context: Studies conducted in “resource-limited settings,” operationally defined as regions where access to and quality of basic healthcare are insufficient due to absolute shortage or inequitable distribution of core medical resources (human, financial, and infrastructural). A setting is considered resource-limited if it meets at least one criterion: The study location is classified as a low-income or lower-middle-income economy by the World Bank; The study focuses on low-income, remote, or marginalized subgroups within upper-middle- or high-income economies; The original text explicitly uses equivalent terms such as “resource-limited” or “low-income” to describe the setting; − The study explicitly reports resource scarcity characteristics such as limited access to healthy food or shortage of primary healthcare workforce.

#### 2.3.2 Exclusion criteria

(1) Studies reporting only physiological and biochemical effects of dietary interventions without any implementation process or determinant data; (2) reviews, editorials, conference abstracts, and study protocols (though their reference lists may be hand-searched); (3) non-Chinese and non-English publications; (4) full-text unavailable or incomplete data; (5) studies conducted among general urban populations in high-income countries without separate analysis of resource-limited subgroups; (6) studies concluding only that “more resources are needed” without presenting empirical evidence of resource constraints.

#### 2.3.3 Operational definition

From the perspective of core health system resource provision, the World Health Organization (WHO) defined resource-limited health contexts in its 2010 handbook Monitoring the Building Blocks of Health Systems: A Handbook of Indicators and Their Measurement Strategies. Such settings are characterized by profound shortages of core health inputs, including workforce, fiscal investment, physical infrastructure, and essential medicines and supplies, which hinder equitable universal coverage of basic clinical care for target populations (6). Quantitatively, the Healthcare Access and Quality (HAQ) Index proposed by the IHME-led Global Burden of Disease (GBD) consortium serves as a validated metric to demarcate resource-constrained geographies, as reported in a 2022 systematic analysis published in The Lancet Global Health. Scaled from 0 (worst performance) to 100 (optimal care), the HAQ Index is calculated via mortality–incidence ratios (MIRs) and risk-standardized death rates (RSDRs) across 32 treatable conditions amenable to timely intervention. Regions with an HAQ score below 50 generally exhibit systemic health resource deficits: low-SDI territories concentrate health investments in infectious disease control, while resources dedicated to non-communicable disease (NCD) dietary management and chronic disease care remain severely underfunded. Consequently, avoidable mortality rates are substantially higher in these regions relative to well-resourced areas with HAQ ≥ 70. In 2019, high-SDI nations recorded a mean HAQ score of 83.4 (95% UI 82.4–84.3), a stark performance gap that substantiates the rationality of the HAQ < 50 threshold for identifying resource-limited settings (7).

Integrating WHO’s qualitative health system framework and the GBD HAQ quantitative benchmark, this study adopts a two-tier definition of resource-limited settings covering two distinct populations: first, low- and middle-income countries (LMICs) per World Bank income classifications; second, marginalized, underserved subpopulations within upper-middle and high-income nations shaped by geographic segregation and socioeconomic inequities in health resource allocation. Four shared defining attributes unify all included resource-scarce contexts: (1) restricted access to affordable nutrient-dense foods; (2) insufficient primary care staff and limited professional nutrition capacity; (3) fragmented infrastructure supporting patient health literacy and chronic disease self-management; and (4) substantial imbalance between population NCD burden and public health investment.

This broad two-tier classification is methodologically justified by prior implementation science research reported by Means et al. (8), who conducted a systematic review of CFIR applications across LMIC contexts. Their work confirmed that contemporary scoping reviews addressing chronic disease implementation routinely integrate both LMIC populations and disadvantaged subgroups within high-income countries; exclusive LMIC-only inclusion would create critical evidence gaps. Indigenous and rural low-income cohorts in high-income jurisdictions face unique barriers such as colonial erosion of traditional food systems and racially inequitable welfare benefit distribution—contextual determinants absent from LMIC literature. Despite divergent root causes of resource deprivation, these two groups share highly homologous barriers to dietary intervention, justifying unified qualitative coding followed by stratified thematic synthesis. Means et al. further recommended that studies encompassing dual resource-limited subgroups must conduct systematic cross-group comparisons to disentangle divergent barriers and enablers: LMICs suffer nationwide systemic underinvestment, whereas health inequities in high-income nations stem from localized uneven resource distribution. To mitigate the heterogeneity introduced by this inclusive definition, the full thematic analysis presented in Section 3.4 adheres strictly to this stratified comparative framework (8).

### 2.4 Literature search strategy

A systematic search across multiple databases combined with supplementary searches was employed. The search timeframe was from database inception to March 2026.

#### 2.4.1 Databases

English databases: PubMed, Embase, CINAHL, Web of Science, Cochrane Library. Chinese databases: CNKI, Wanfang Data, VIP Journal Database. Grey literature: Google Scholar, OpenGrey, China Important Conference Papers Full-text Database, and official websites of relevant organizations.

#### 2.4.2 Search terms

Search terms were constructed around four core conceptual groups: type 2 diabetes mellitus, diet/nutrition, implementation/barriers/adaptation, and resource-limited settings. The full reproducible electronic search syntax of PubMed is provided in Supplementary Table S1 for replication reference.

### 2.5 Study selection and data extraction

#### 2.5.1 Selection process

Two reviewers independently conducted study selection. EndNote X9 was used for literature management and deduplication. Titles and abstracts were initially screened to exclude obviously irrelevant records; full texts were then obtained for eligibility assessment. Inter-reviewer agreement was required to exceed 90%; disagreements were resolved through discussion or adjudication by a third reviewer (supervisor).

#### 2.5.2 Data extraction

A pre-designed standardized form was used to extract data, including: - Basic study information (author, year, country, study design, objectives); − Population characteristics (sample size, ethnicity, urban/rural distribution, socioeconomic status); − Intervention characteristics (dietary strategy type, implementer, intervention duration, follow-up time); CFIR-related data (all reported barriers, facilitators, adaptation strategies, implementation outcomes, with verbatim quotations and page numbers); Research gaps (limitations and future directions explicitly stated in the discussion).

### 2.6 Data items

Two sets of data items were predefined for charting: basic study metadata and CFIR 2.0 coding variables covering five core implementation domains. All extracted variables were strictly defined based on the official CFIR 2.0 manual. During thematic synthesis, identical barrier and facilitator themes from different studies were merged to simplify analysis, and no extra subjective assumptions were introduced into data extraction.

### 2.7 Data analysis and synthesis

#### 2.7.1 CFIR coding procedure

We used the 2022 updated version (CFIR 2.0) for deductive coding, which expanded the original constructs to better capture context-specific factors, especially in resource-limited settings. After training and pilot coding calibration, two reviewers independently mapped verbatim statements of barriers/facilitators to the most matching CFIR constructs. Unmappable text was open-coded and categorized through team discussion.

#### 2.7.2 Synthesis strategy

A mixed quantitative-qualitative synthesis was performed: (1) descriptive statistics of basic study characteristics (country, year, study design); (2) thematic narrative synthesis to summarize second-level themes within each CFIR domain, supported by representative verbatim quotes; (3) cross-tabulation analysis to construct a barrier-facilitator correlation matrix and an evidence gap map, with heatmaps visualizing density and gaps across CFIR domains, geographic regions, and population subgroups.

### 2.8 Quality appraisal

Consistent with scoping review methodology, formal risk of bias assessment was not performed. However, to assist in interpreting evidence strength, study designs and reporting completeness were graded and described.

## 3 Results

### 3.1 Study selection process and results

**Figure 1:**
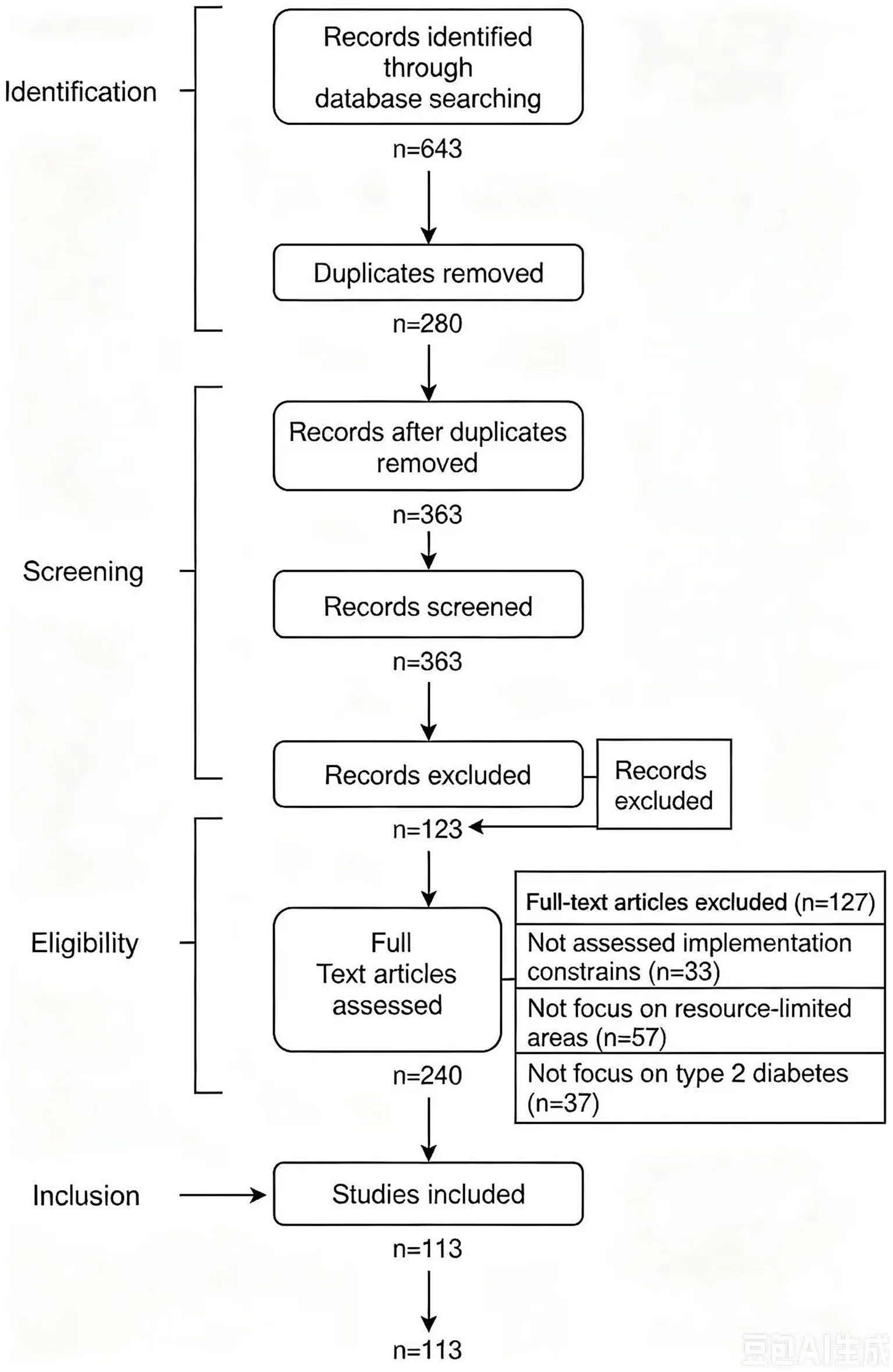
A total of 643 records were retrieved from electronic databases. After removing 280 duplicates, 363 unique records underwent title and abstract screening, where 123 irrelevant articles were excluded. The remaining 240 full-text papers were assessed for eligibility, and 127 articles were excluded for three primary reasons: failure to assess implementation barriers and facilitators (*n* = 33), non-resource-limited research settings (*n* = 57), and irrelevant focus unrelated to type 2 diabetes management (*n* = 37). Finally, 113 eligible studies were included for thematic synthesis under the CFIR framework.

The initial search yielded 643 records. After deduplication, 363 records remained. Following title and abstract screening, 123 records were excluded. The remaining 240 full-text articles were assessed for eligibility, and a further 127 were excluded for three distinct predefined reasons: 33 articles failed to assess implementation barriers and facilitators, 57 articles were not conducted in resource-limited settings, and 37 articles did not focus on type 2 diabetes mellitus. A total of 113 studies were finally included. The study selection process is shown in Figure 1. PRISMA-ScR flow diagram illustrating the literature screening and selection process for this scoping review on evidence-based dietary strategy implementation for type 2 diabetes mellitus management in low- and middle-income countries.

### 3.2 Basic characteristics of included studies

A total of 113 eligible studies were included in this scope review (9–121). These studies were published between 2002 and 2025, with an overall increasing trend in publication volume. Geographically, studies covered global regions, mainly distributed in Asia (28 studies, 24.78%) (9, 10, 24, 32, 35–39, 46, 52, 53, 55, 56, 59, 62, 64, 91, 97, 99–107), North America (28 studies, 24.78%) (10, 17–19, 22, 28–31, 33, 34, 44, 48–51, 60, 61, 69, 72, 74, 76, 79, 84, 86, 92–94), and Africa (25 studies, 22.12%) (12, 14–16, 20, 25, 27, 42, 43, 45, 57, 58, 63, 66, 67, 70, 75, 77, 82, 83, 87, 88, 90, 96, 98). The most frequently represented countries were the United States (27 studies) (10, 17–19, 22, 28–31, 33, 34, 44, 48–51, 60, 61, 69, 72, 74, 76, 79, 84, 92–94), China (18 studies) (32, 35, 36, 39, 46, 56, 59, 64, 97, 99–107), and South Africa (8 studies) (14, 15, 43, 45, 58, 63, 90, 96). Regarding study settings, urban areas (38 studies, 33.63%) (10, 12, 13, 17, 21, 26, 31, 35, 38, 46, 48–50, 54, 55, 63, 64, 73, 75, 77, 78, 80, 87, 97–110, 112, 116) and rural areas (34 studies, 30.09%) (18, 19, 22, 23, 28–30, 34, 44, 47, 51, 53, 56, 60, 67, 69, 70, 74, 76, 79, 81, 84, 86, 88, 90, 91, 111, 113–115, 117, 118, 120, 121) accounted for the majority.

Thirty-six studies (31.86%) (9, 11, 14–16, 20, 24, 25, 27, 32, 33, 40–43, 45, 52, 57–59, 61, 62, 65, 68, 71, 72, 82, 83, 85, 89, 93–96, 105, 119)used a combined urban–rural setting, and 5 studies (4.42%) (36, 37, 39, 66, 92) did not specify urban/rural status.

The four most dominant study designs accounted for the majority of all included papers: Cross-sectional studies (*n* = 25) (27, 31, 39, 40, 45, 47, 48, 62, 65, 67, 71, 74, 76, 79, 85, 87, 98, 100, 101, 105, 108, 113, 114, 116, 119); randomised controlled trials (including parallel RCTs, cluster RCTs, pilot RCTs, *n* = 24) (17, 18, 25, 35, 37, 44, 46, 55, 64, 69, 80, 81, 84, 89, 99, 102–104, 107, 109, 112, 118, 120, 121); qualitative studies (*n* = 20): (12, 20, 21, 28, 33, 34, 36, 41, 42, 50, 54, 57, 60, 63, 70, 73, 75, 77, 82, 86); mixed-methods studies (*n* = 18) (13, 16, 32, 38, 43, 49, 51, 58, 72, 88, 90, 93–95, 97, 106, 110, 111).

Other less frequent designs contained quasi-experimental studies, observational cohort/case–control research, formative studies, case-based papers, expert seminar reports and economic modelling research. In total, 113 eligible studies were included in this scoping review. Full detailed characteristics of each included primary study are summarized in Supplementary Chart S1, where readers can access complete individual-level information for all eligible articles.

### 3.3 Co-occurrence network analysis of implementation barriers and facilitators

**Figure 2:**
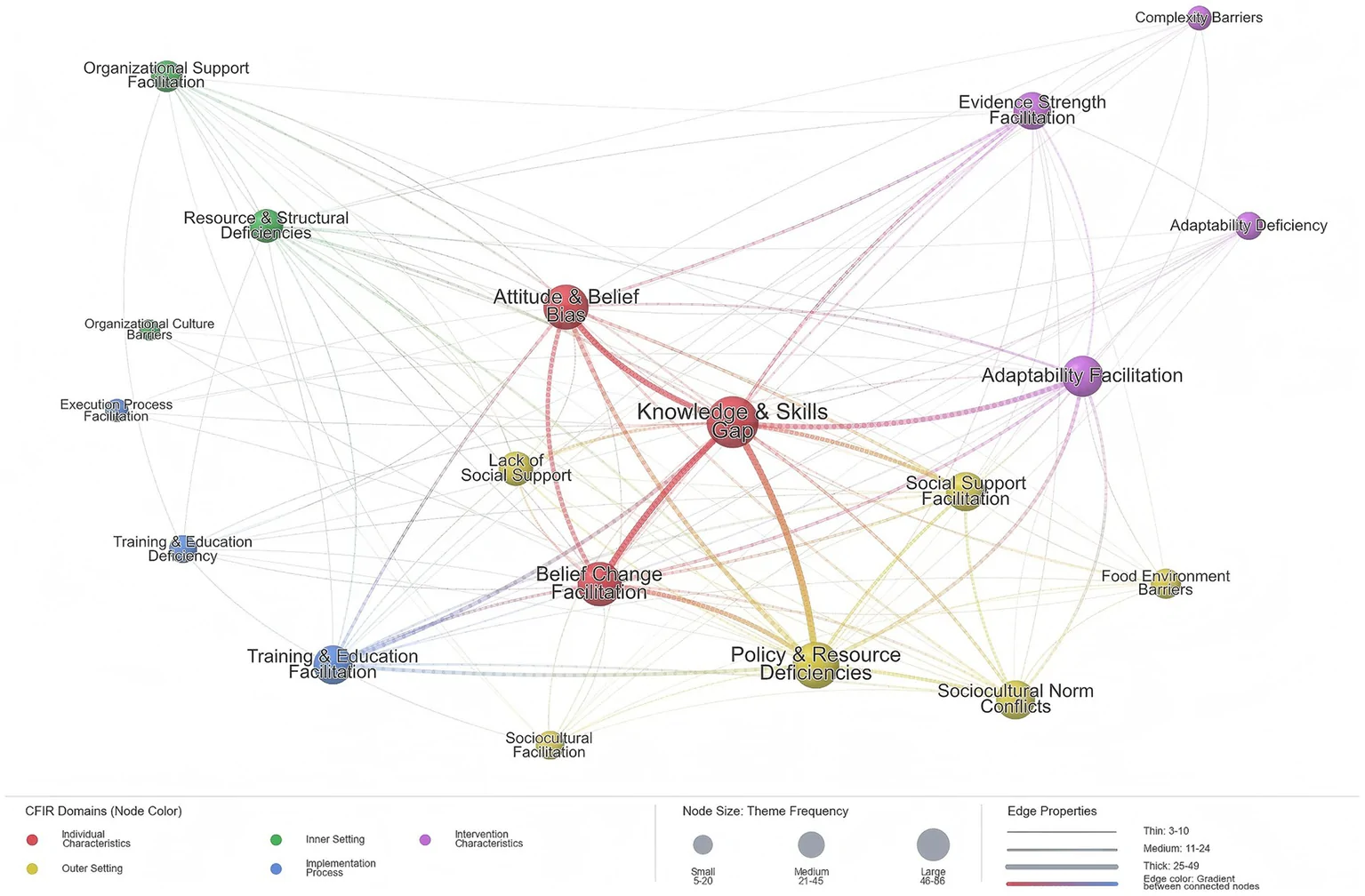
Network visualisation of CFIR-based barriers and facilitators to dietary diabetes management interventions in low- and middle-income countries. Co-occurrence network of implementation themes based on the Consolidated Framework for Implementation Research (CFIR 2.0). Node color represents the five CFIR domains: Individual Characteristics (red), Inner Setting (green), Intervention Characteristics (purple), Outer Setting (yellow), and Implementation Process (blue). Node size reflects theme frequency across included studies (small: 1–20 studies; medium: 21–60 studies; large: >60 studies). Edge thickness indicates co-occurrence frequency (thin: 1–10; medium: 11–40; thick: >40 co-occurrences). Edge color shows a gradient between connected nodes.

A co-occurrence network analysis was conducted to visualize interrelationships among barriers and facilitators across the five CFIR domains (Figure 2).

### 3.4 CFIR thematic coding results of dietary management barriers and facilitators

This study systematically coded and classified barriers and facilitators for dietary self-management in diabetic patients using the Consolidated Framework for Implementation Research (CFIR), covering five core domains: Individual Characteristics, Outer Setting, Inner Setting, Intervention Characteristics, and Implementation Process. A total of 113 eligible publications were included for thematic analysis. Table 1 summarizes the second-order themes alongside representative empirical evidence corresponding to each CFIR dimension.

**Table 1:** CFIR-based thematic synthesis of barriers and facilitators to evidence-based dietary strategies for type 2 diabetes.

First-level dimension	Second-level theme	Frequency (*n* = 113)	Representative citations
Individual characteristics	Attitudes and belief biases (9–11, 16, 17, 20–23, 26, 28–34, 36–40, 42, 45, 46, 48, 50, 52, 54, 55, 57, 60–62, 64, 65, 73, 74, 76–78, 83, 85, 86, 88, 89, 92, 99, 100, 103, 107, 109, 110, 112, 113, 115–118, 121)	60/113	In peri-urban Kenyan communities, residents demonstrate flawed baseline health beliefs featured by low perceived susceptibility to diabetes, limited recognition of the benefits brought by dietary adjustments and poor nutrition self-efficacy; inadequate diabetes-related knowledge renders residents susceptible to misleading health information and unhealthy dietary decisions (11). In Cambodia’s primary healthcare context, the absence of Khmer-adapted patient educational materials leads to widespread public misunderstanding of evidence-based glycaemic control dietary advice, forming biased attitudes that hinder sustained adherence to healthy lifestyle regimens (10). For Australian Aboriginal groups, colonisation-induced breakdown of intergenerational traditional food knowledge creates cultural belief biases, generating dissonance between mainstream low-carb diabetes guidance and community food values and reducing participants’ willingness to follow metabolic remission dietary plans (9).
	Knowledge and skill deficits (10, 14, 15, 17, 18, 21–25, 27, 31, 32, 35–44, 46–50, 52–57, 59, 60, 63–77, 79–90, 92–94, 96–99, 101–107, 109–117, 119–121)	85/113	Among American Indian and Alaska Native adults living with type 2 diabetes, multi-group stakeholder interviews revealed widespread practical skill gaps, including inadequate ability to judge standard food portion sizes, insufficient cooking skills for fresh fruits and vegetables, and a lack of time-efficient meal preparation strategies. Many participants avoided purchasing fresh produce due to worries about spoilage resulting from poor cooking know-how, which generated frequent food waste and pushed them to rely heavily on high-processed convenience foods (19). In rural South African villages, untrained home-based carers demonstrated severe diabetes knowledge deficits prior to formal standardized training; the majority were unable to provide precise dietary counselling, identify hypoglycaemic manifestations, assist patients with insulin administration, or deliver targeted advice on balanced staple intake and portion management (76).
	Belief transformation promotion (10, 13, 16, 22, 27, 29–53, 56–58, 62, 63, 67–69, 73, 76–78, 82, 86, 88–92, 95, 98, 100–102, 105, 107–114, 118, 121)	52/113	Standard Khmer vernacular educational booklets developed for primary care clinics deliver systematic dietary and metabolic knowledge to rectify fragmented, misleading lay health perceptions, gradually reshaping patients’ biased understandings of glycaemic management and cultivating positive attitudes toward long-term self-care (10). Meanwhile, structured peer support groups serve as powerful catalysts for transforming negative diabetes-related beliefs among rural Chinese patients (104). Through regular group discussions, shared disease experience and mutual emotional encouragement, participants gain a stronger sense of identity and belonging, relieve anxiety and depression caused by chronic disease, and abandon pessimistic, passive attitudes toward treatment and diet control (104). Compared with one-way routine nurse-led education, peer interactive communication helps patients rebuild rational cognition of diabetes self-management, recognize the tangible benefits of sustained diet and exercise intervention, and convert negative health beliefs into proactive, consistent self-care behaviours (104).
Outer Setting	Food environment barriers (9, 11, 14–18, 20, 22, 24, 33, 54, 58, 59, 61, 67, 79, 86, 90)	18/113	Multiple structural food environment barriers undermine consistent diabetic dietary adherence among rural and Indigenous populations, creating persistent obstacles to accessing balanced, glycaemia-friendly meals. For rural American Indian reservation communities, severe geographic food deserts represent a core environmental constraint: the nearest full-service supermarket can be nearly 90 miles away, while local small convenience and dollar stores only stock limited, low-quality fresh produce at inflated prices, discouraging regular purchases of fruits and vegetables critical for blood sugar control. Transportation deficits compound this barrier, as most community members lack reliable private vehicles and there is no public transit system to reach distant grocery retailers, forcing reliance on shelf-stable, high-sodium commodity foods distributed via federal food assistance programmes. Even with government food subsidies such as SNAP and FDPIR, monthly fixed allotments and short fresh produce shelf lives lead participants to prioritize ultra-processed convenience foods over whole-grain, low-sugar options to avoid food waste (14). Compounding physical food access limitations, rural diabetic individuals confront digital food environment barriers when adopting telehealth self-management tools; large proportions of older adults and low-income rural residents lack smartphones, steady internet access and basic digital literacy to operate diet-tracking mobile software, rendering app-based food logging unworkable and forcing cumbersome paper-based dietary recording as an alternative (15).
	Sociocultural norms conflicts (9–14, 16, 18, 19, 21, 23, 25–28, 34, 37–39, 47, 50, 54, 57, 58, 62, 63, 66, 67, 72, 73, 75, 77, 81, 87, 91, 94, 98, 102, 104, 105)	40/113	In Cambodia’s primary care context, standardized Western-style diet guidance embedded in national chronic disease toolkits fails to align with local cultural eating customs; generic low-sugar, low-carb recommendations overlook staple carbohydrate-based traditional meals widely consumed by residents, creating normative dissonance that weakens patients’ willingness to adopt clinical dietary advice (10). For rural American Indian reservation communities, long-standing disruption of Indigenous traditional food systems driven by colonial policies generates profound sociocultural norm conflict: modern evidence-based diabetic nutrition guidance prioritizing low-processed produce clashes with community cultural identities centered on historic hunted, gathered and cultivated Indigenous foods, while federal commodity food programs distribute shelf-stable processed goods that conflict with both ancestral dietary norms and glycaemic management requirements (19). Among type 2 diabetes patients in Northern Ethiopia, deeply entrenched cultural eating routines featuring large portioned communal meals, regular high-starch staples and limited separation of healthy and indulgent food customs directly contradict structured portioning, calorie-limiting and balanced meal norms promoted in clinical nutrition counselling; even patients receiving formal nutrition education struggle to decouple lifelong sociocultural eating habits from evidence-based diabetic diet rules, resulting in overwhelmingly poor overall dietary adherence (13).
	Policy and resource shortages (11–13, 15–20, 22–25, 27–30, 33, 34, 36, 38, 40, 41, 43–47, 49–57, 60, 62, 63, 65, 67, 68, 73–77, 79, 80, 83, 84, 86, 88, 96, 98, 111, 112, 119, 120)	65/113	In Middle Eastern and African healthcare systems, incomplete national noncommunicable disease policies fail to allocate sufficient funding for sustained nutrition education, affordable diabetic food subsidies, and widespread training of specialized diet educators; limited government healthcare expenditure creates shortages of testing supplies, low-cost anti-diabetic medications, and culturally tailored patient educational materials, while uneven distribution of medical staff widens care disparities between rural and urban populations (3). Rural U.S. community extension programs also face persistent resource constraints: though hands-on diabetes cooking curricula can effectively improve healthy eating behaviours, limited county budgets restrict educator training frequency, reduce program scale, and limit the provision of free low-sugar, low-sodium recipe resources and multilingual handouts for ethnic participants (126). In Bangladesh’s primary care context, weak public health financing policies result in extremely high out-of-pocket medical costs for diabetic households; insufficient state subsidies for fresh nutritious foods, combined with inadequate investment in mobile health infrastructure, leave many low-income patients unable to afford balanced meals or access regular remote lifestyle reminders that support diet adherence (24).
	Sociocultural facilitators (3, 9, 19, 20, 22, 40, 45, 59, 66, 70, 72, 77, 79, 102, 122)	15/113	In multi-ethnic deprived communities in England, trusted local VCSE services deliver hyper-local, culturally tailored diabetes support including ethnic language nutrition sessions, culturally appropriate recipe sharing and long-term peer group gatherings; these community embedded structures build a sense of belonging, bridge gaps between patients and formal NHS education, and provide sustained informal guidance that mitigates emotional distress after diabetes diagnosis, enabling participants to gradually adjust cultural eating habits aligned with glycaemic targets (40). In rural Bangladesh, participatory learning and action (PLA) community groups function as powerful sociocultural enablers by setting up gender-inclusive discussion circles, organising village collective meetings and partnering with local religious leaders such as imams to integrate diabetes lifestyle advice into daily cultural communication; community-led kitchen gardening collectives and small mutual aid funds also ease financial barriers to fresh produce, while shared group exercise norms reduce social stigma against women’s outdoor physical activity, jointly boosting compliance with healthy dietary practices (77). For American Indian and Alaska Native communities, reviving traditional Indigenous food culture serves as a critical sociocultural facilitator; centering ancestral hunted, fished and farmed ingredients in nutrition education reconciles tribal cultural identity with diabetes dietary rules, and community stakeholder coalitions combining elders, nutrition educators and tribal leaders co-design context-fit eating guidance that respects long-standing sociocultural food customs, making balanced low-glycaemic diets more culturally acceptable and sustainable (19).
	Insufficient social support (4, 21, 27, 28, 30, 35, 36, 39, 49, 52, 54, 56, 58, 61, 64, 67, 68, 79, 80, 90, 93, 96, 98, 99, 104, 126, 129)	27/113	For rural Chinese adults with type 2 diabetes, inadequate objective family assistance, scarce subjective emotional comfort and low utilisation of available social resources significantly impair their compliance with dietary rules; patients with low overall social support show far poorer performance in following regular meal timing, carbohydrate counting and holiday dietary restrictions, while severe depressive emotions easily arise without family reminders and psychological comfort to sustain long-term diet control (99). In rural multiethnic Texas communities, participants often struggle to maintain consistent diabetic meal plans due to a lack of household support: many families prepare separate regular dishes rather than unified low-sugar, low-fat meals for diabetic members, and limited peer communication channels leave patients without shared experience exchange and mutual supervision on healthy cooking and portion management (126).
	Facilitative social support (1–5, 8, 12, 16, 18, 22, 27, 28, 34, 40, 44, 46, 48, 49, 51, 52, 55, 56, 59, 61, 63, 69, 72, 74, 84, 86, 87, 92, 97, 99, 100, 103, 122, 124, 125, 128)	40/113	In peri-urban Kenyan settlements, culturally adapted diabetes group courses run by community health workers deliver persistent informational and motivational social support; five consecutive days of offline interactive learning sessions plus 4 weeks of follow-up mobile messages significantly raise participants’ perceived diabetes susceptibility, perceived benefits of dietary modification and nutritional self-efficacy, eliminating psychological obstacles and driving reduced consumption of refined grains and cooking oil alongside higher fruit intake (11). For adults with type 2 diabetes living in remote northern Chinese urban areas, standardized hospital-led nutritional education constitutes formal supportive resources: systematic nutrition lectures, one-on-one personalized meal planning coaching and monthly telephone follow-ups enrich patients’ dietary knowledge, while involving family members in nutrition counselling delivers stable daily supervision and emotional support. The integrated professional and familial support system greatly lifts participants’ mastery of food exchange systems and balanced meal arrangement, and yields statistically significant declines in fasting and postprandial blood glucose (18).
Inner Setting	Resource and structural inadequacies (9, 11, 14, 15, 18, 20, 24–26, 29, 35, 45, 49, 51, 63, 68, 71, 75, 77, 80, 83, 84, 91, 96, 98, 118)	26/113	1. Multiple structural and resource deficits undermine consistent diabetes self-management for Ngarrindjeri Aboriginal community members living in remote regional areas. Geographical isolation restricts frequent in-person nutrition support, and local primary health services lack dedicated continuous glucose and ketone monitoring tools as well as culturally adapted dietary guidance materials. Routine public health service models do not deliver ongoing group nutrition sessions and sustained health coaching, while limited access to affordable fresh produce creates practical barriers to following recommended low-carb dietary approaches (9). 2. Low- and middle-income country primary healthcare systems are constrained by structural medical shortages that impede systematic diabetes nutrition counselling, including insufficient health workforce quantity and expertise, inadequate diagnostic screening equipment, and a scarcity of standardized, up-to-date chronic disease educational resources (11). 3. Multiple structural and resource inadequacies in Ghana’s primary healthcare system and local living environments hinder consistent diabetic dietary self-management. System-level constraints include insufficient trained clinical workforce, scarce dedicated dietitians, limited formal nutrition training for general providers, and compressed consultation time that restricts detailed dietary guidance. Clinically, persistent shortages of glucometers and expensive testing strips impede routine blood glucose monitoring. In terms of educational resources, mainstream diabetes pamphlets are not culturally tailored to local staple foods, lacking practical indigenous meal guidance. Beyond medical infrastructure, seasonal erratic supply of fresh produce and widespread household financial hardship create material barriers to maintaining balanced low-carb diets, while long travel distances to hospital clinics further cut off sustained nutrition follow-up support (68)
	Organizational support facilitators (20, 24, 28, 30, 32, 33, 36, 44, 51, 61, 64, 66, 68, 70, 79, 80, 82, 83, 85, 87, 97)	21/113	1. Organized medical support acts as a key facilitator for older adults Bai patients’ diabetes self-management. Hospitals provide multidisciplinary professional guidance, culturally tailored health education classes and standardized step-by-step intervention procedures. Integrated medical alliance links township clinics to deliver accessible, bilingual diabetes counselling for rural seniors, while systematic physical examination reminders and hands-on operation training further strengthen patients’ long-term dietary and medication adherence (97). 2. Limited organizational enablers for diabetic dietary management exist within local tertiary hospitals. Dedicated weekly diabetes clinics provide stable institutional platforms where nurses, physicians and full-time nutrition staff deliver personalised dietary counselling to patients. Informal internal case conferences among care teams also allow providers to exchange practical nutrition guidance, supporting consistent self-care advice for type 2 diabetes patients (68) 3. Formal hospital diabetes care systems constitute core organizational facilitators for type 2 diabetes self-management. Clinical nurses and clinicians deliver continuous dietary and physical activity counselling during routine outpatient visits, and they also remind patients of healthy lifestyle rules when encountered in community public spaces. Urban public hospitals sustain structured diabetes peer support groups led by retired nurses, creating a regular institutional platform for patients to exchange practical diet and exercise self-management experience. Such hospital-based professional guidance and peer group systems form stable organizational support to improve patients’ long-term adherence to recommended healthy behaviours (28)
	Organizational and cultural barriers (21, 36, 66, 76, 102)	5/113	1. Remote rural islands suffer severe shortage of diabetes specialists, dietitians and professional self-management educators, leading to insufficient specialized chronic disease services (60). 2. Healthcare system dependency: reliance on MSF (Médecins Sans Frontières) support; the local health department lacks ownership and sustained investment, raising concerns about project sustainability(p7-p8) (70) 3. Misaligned treatment priorities: antihypertensive and statin medications cost less than sulfonylureas or insulin, yet glucose-lowering drugs are more commonly used in practice, while lipid management is neglected (p20) (96)
Intervention characteristics	Poor adaptability (Barrier) (10, 41, 43, 45, 51, 73, 84, 87, 93, 95, 115, 117, 120, 121)	14/113	1. The SMS-based diabetes intervention shows obvious poor contextual adaptability as a digital self-management tool. First, national low literacy rates create comprehension barriers for patients to grasp professional diet and glycaemic knowledge delivered via brief standardized text messages. Second, one-size-fits-all generic SMS content is not tailored to local Bangladeshi daily eating customs, religious lifestyles and diverse household economic conditions, lacking individualised lifestyle guidance. Third, uneven mobile ownership, shared household phones and unstable communication infrastructure hinder consistent message reception, making the intervention unsuitable for low-resource groups without private digital devices (41) 2. Low calcium and vitamin D dietary patterns represent a critical adaptive barrier for Chinese female patients with type 2 diabetes. The traditional Chinese grain-and-vegetable-based diet lacks dairy and calcium-rich foods, making it hard for women to reach protective calcium intake levels; postmenopausal women face aggravated bone metabolism defects and insulin resistance due to long-term insufficient calcium and serum 25OHD, further weakening the response to standard diabetic diet and glycaemic control interventions. Additionally, most diabetic females reduce intake of calcium-containing animal foods after diagnosis, which creates persistent incompatibility between routine diabetes dietary guidance and their actual long-term eating habits (45) 3. Primary diabetes care in Cambodia faces prominent contextual poor adaptability barriers. Standard international chronic disease management guidelines and generic lifestyle education materials lack deep localisation to fit Cambodian staple food culture and rural living contexts. Though the newly developed toolkit contains basic diet advice leaflets translated into Khmer, these universal lifestyle materials do not provide customised meal plans, region-specific low-carb recipes or culturally tailored dietary operation guidance based on local traditional ingredients. Primary health facilities only hold general diet suggestions without context-adapted counselling tools, creating incompatibility between standard diabetes dietary advice and residents’ long-term indigenous eating habits (10)
	Evidence-based strength (Facilitator) (9, 15, 23, 26, 37, 43, 45, 46, 55, 56, 61, 62, 69, 71, 78, 87, 89, 94–97, 99, 102–104, 106, 107, 109, 110, 112, 115–118, 120, 121)	36/113	1. Low-carbohydrate dietary intervention carries robust clinical evidence and serves as a key evidence-based enabler for Aboriginal T2D remission programmes. Multiple systematic reviews and the official clinical consensus statement from the Australian Diabetes Society confirm carbohydrate-restricted nutrition therapy delivers meaningful improvements in HbA1c, insulin resistance and lipid profiles, and even enables medication-free type 2 diabetes remission. This intervention is underpinned by abundant international RCT evidence and established behavioural change frameworks, forming a formal guideline-endorsed clinical tool for community-led Indigenous chronic disease care. Beyond universal clinical evidentiary support, the low-carb framework was refined via Aboriginal co-design workshops to boost local cultural suitability, further strengthening its implementability among Ngarrindjeri Aboriginal residents (9) 2. This diabetes nutrition intervention gains solid evidence support from the Health Belief Model and global diabetes guidelines. Cluster randomised trial data prove the culturally tailored educational programme effectively lifts participants’ diabetes awareness, nutrition self-efficacy and reduces intake of unhealthy refined grains and cooking oils, forming a theory-backed, implementable self-management tool for peri-urban African populations (11)
	Adaptability promotion (Facilitator) (9, 12, 15–19, 24, 29, 35, 38, 43, 44, 47, 48, 52–54, 57, 58, 61, 63, 64, 66, 72, 73, 75, 78, 80, 81, 84, 86, 89–95, 97, 102, 105, 106, 110–112)	46/113	1. Co-designed, culture-centred low-carb intervention acts as a key adaptability enabler. Developed via Aboriginal elder workshops, the programme integrates Indigenous food knowledge and creates custom meal guides, recipe resources and weekly ingredient boxes fitting local traditional food access. It combines Aboriginal yarning-style counselling with standard diabetes management tools to align intervention design with community cultural norms, greatly boosting the acceptability and sustainability of dietary self-management among Ngarrindjeri Aboriginal residents (9) 2. Stage-tailored, individualized intervention planning serves a critical adaptability facilitator. Based on the Transtheoretical Model, clinicians can assess patients’ readiness to change diet first and deliver differentiated counselling and targeted problem-solving guidance matching their behavioural stages. This person-centred matching reduces perceived dietary barriers and strengthens diabetes self-efficacy, which significantly boosts long-term dietary adherence among low-income underserved diabetic populations (17) 3. Locally tailored mobile voice reminder system acts as a strong adaptability enabler for diabetic self-management. The m-health intervention delivers personalised health guidance in native Bengali language via voice calls, avoiding literacy barriers caused by text messages. All reminder contents are customized to individual patients’ medication plans, dietary needs and exercise conditions, and patients can access free 24/7 physician consultation hotlines anytime to solve real-time lifestyle difficulties. Such language-friendly, individualised remote support greatly raises the usability and long-term adherence of diabetes self-management guidance among low-literacy Bangladeshi patients (24)
	Complexity-related barriers (26, 29, 56, 69, 78, 80, 87, 90, 92)	9/113	1. Multi-layered self-management requirements form prominent complexity barriers for Iranian type 2 diabetic patients. Weight loss intervention relies on integrated diet adjustment, regular exercise and long-term medication compliance simultaneously, which imposes heavy cognitive and behavioural burdens on patients. Multiple interrelated obstacles coexist: inadequate dietary decision-making capacity, frequent emotional eating temptations, and diverse exercise barriers including fatigue, pain and limited public sports facilities. Besides, demographic and disease variables compound intervention complexity — older adults, low-literacy, rural and long-duration diabetic patients face overlapping self-care difficulties, making integrated lifestyle modification hard to sustain (26) 2. Confusing, inconsistent dietary guidance creates major complexity barriers for Black South African type 2 diabetes patients. Primary care staff lack systematic nutrition training and deliver conflicting food advice; some clinicians forbid staple maize porridge while others permit it, leaving patients uncertain about allowed foods and portion standards. Few participants receive standardized food exchange charts, and most cannot grasp accurate portion control rules without clear measuring guidance. In addition, patients receive fragmented advice without integrated cultural, economic and daily living considerations, forcing them to struggle to reconcile clinical diet rules with local traditional food customs, funeral feasts and affordable household ingredients, which greatly reduces sustained dietary adherence (29) 3. Standard diabetes dietary guidance creates severe implementation complexity for conflict-affected Congolese patients. The recommended healthy foods are unaffordable and scarcely available locally, forcing patients to struggle to separate special diabetic meals from household regular diets and bear extra economic burden. Meanwhile, the multi-layered management burden compounds complexity: patients must combine strict diet control, regular long-distance clinic trips amid regional unrest and consistent medication intake; food shortages often lead them to skip medicines, forming a vicious cycle of unmanageable self-care demands. Additionally, local communities hold misinformed perceptions of diabetes as a curable rich people’s illness, and many rely on traditional healers rather than following complicated long-term chronic care regimes (56)
Implementation process	Training and education deficits (10, 13, 21, 27, 30, 32, 42, 43, 45, 51, 54, 71, 83, 114)	14/113	1. Primary care staff in Cambodia face notable training shortages for non-communicable disease management. Local health centres have limited prior experience delivering standardized diabetes and hypertension services; there is no systematic, sustained training curriculum to support frontline workers in mastering multi-step screening, risk assessment and referral workflows aligned with international NCD guidelines. While the locally adapted Khmer-language toolkit was designed to fill operational gaps, formal supporting training manuals and regular refresher sessions for clinic personnel remain absent, leaving grassroots providers unable to fully and accurately execute complex evidence-based care protocols, which generates inconsistent diabetes management quality across facilities (10)
	Execution process facilitators (18, 52, 55, 81, 84, 95, 99)	7/113	1. Multi-layered interactive SMS follow-up forms core execution enablers to sustain participant engagement. The digital SMART system delivers personalised weekly text modules tailored to users’ self-prioritised health goals, with two-way reply triggers and automated encouraging auto-replies to reinforce healthy behaviour. Proactive follow-up protocols are embedded: auto reminder messages are sent for non-response after 3 h, and research staff conduct outbound phone calls if users fail to reply for three consecutive days to resolve technical or motivational barriers. Short, plain-language content with supplementary hyperlinks lowers comprehension burden, while flexible message delivery times match participants’ daily schedules, jointly maintaining steady response rates and self-management motivation (81) 2. Multi-channel regular follow-up and individualized paper diet plans act as core implementation enablers for dietary behaviour modification. The intervention adopts layered education delivery, including group lectures on GI/GL knowledge and one-on-one customized recipe design based on patients’ daily activity and food preferences. Weekly telephone follow-up is arranged to track dietary records, correct unreasonable eating habits, and provide timely targeted guidance on fruit intake, sugar limitation and food portion management. Uniform 3-day dietary record notebooks are distributed to standardize self-monitoring, while printable GI/GL food tables and food exchange lists simplify daily food selection operations, lowering the operational difficulty of long-term dietary adherence (95) 3. Culturally adapted offline group intervention tools and multi-layered on-site support constitute core implementation enablers for dietary and physical activity behaviour change. The program converts abstract international diabetes guidelines into pictorial French PowerPoint materials and simplified paper tracking logs tailored for low-literacy local residents, replacing complex calorie counting with intuitive staple portion recording rules. Structured 14-session offline group courses integrate role-play, food cooking demonstrations and neighborhood exercise group practice; community health workers conduct regular home visits between sessions to follow up participants’ daily diet and workout implementation. In addition, culturally matched low-cost recipe booklets for local traditional sauces and at-home workout guide handouts lower operational barriers, while family participation guidance embedded in each session mobilizes household cooking partners to jointly maintain healthy eating habits, significantly lifting long-term lifestyle adherence (52)
	Training and education promotion (11, 14–19, 22, 23, 25–28, 31, 32, 34, 35, 37, 38, 40, 43, 46, 47, 49, 50, 53, 56, 60, 63, 64, 81–83, 86, 87, 92, 93, 98, 109, 110, 114)	40/113	1. Theory-driven, culturally tailored group health education delivered by trained community health workers serves a core facilitator for diabetes dietary and metabolic management. All educational sessions adopt low-literacy teaching strategies including plain local language, large-font visual charts, food demonstrations and teach-back verification to lower comprehension barriers. The intervention adopts modular curriculum covering nutrition, staple food selection and portion control; after offline face-to-face courses, weekly personalised WhatsApp/SMS text reminders are continuously delivered to consolidate dietary knowledge and reinforce healthy eating behaviours. Systematic pre-service training equips community health workers with standardized behavioural guidance skills rooted in the Health Belief Model, enabling them to deliver targeted counselling and raise participants’ disease awareness, nutrition self-efficacy and long-term dietary adherence, ultimately reducing refined grain and oil intake and optimising glycaemic indicators (11) 2. Stage-tailored diabetes dietary education and targeted psychological counselling serve core facilitators to boost patients’ self-management willingness and adherence. Healthcare providers can deliver differentiated education matching individuals’ readiness to change under the transtheoretical model (TTM): for patients in contemplation and preparation stages, focused guidance shall be provided to reduce perceived dietary barriers; those entering the action stage need systematic training on carbohydrate counting, portion control and dietary problem-solving skills. Standardized brief assessment tools (PDQ, DSES, DKT) help clinicians quickly identify patients’ knowledge gaps and behavioural obstacles, so as to adjust educational focus dynamically. Enhancing diabetes self-efficacy via skill-oriented education can effectively lower perceived diet-related difficulties, promote long-term healthy eating behaviours and optimise glycaemic management outcomes. Notably, male patients and low-income underserved groups require intensified, repeated dietary education to improve their engagement with self-management plans (17) 3. Targeted dietary education guided by bone metabolism and glycaemic indicators is an effective intervention measure for Chinese female type 2 diabetes patients. Current Chinese residents generally have insufficient calcium intake due to grain-based traditional eating patterns with limited dairy consumption, which elevates diabetes risk especially for postmenopausal women. Clinical nutrition education should combine blood 25-hydroxyvitamin D and serum calcium test results to deliver personalized dietary guidance: prioritize popularizing high-calcium food intake advice (dairy products, bean food) for female patients, appropriately adjust total energy and fat intake plans, and balance carbohydrate supply to reduce postprandial blood glucose spikes. Differentiated nutrition education by gender is required, as calcium and vitamin D supplementation only shows protective effects on women’s glycaemic control, while male patients need focus on limiting excessive alcohol and total energy intake. Standardized nutritional knowledge popularization can correct unbalanced daily dietary structures, raise awareness of bone-nutrition-glycaemia interactions, and support long-term blood glucose management (45)

#### 3.4.1 Individual characteristics

Three subthemes were extracted within the Individual Characteristics domain, which reflect intrinsic patient-level facilitators and constraints for adherence to diabetic dietary regimens.

##### 3.4.1.1 Attitudes and belief biases (barrier)

Distorted health perceptions and inadequate health awareness constitute core attitudinal and cognitive biases, serving as key individual-level barriers to stable diabetes self-management among low- and middle-income populations and Indigenous ethnic groups (23–25). In peri-urban Kenyan communities, residents hold flawed baseline health beliefs, including underestimated personal diabetes susceptibility, insufficient recognition of dietary modification benefits, and poor nutritional self-efficacy. Deficient diabetes literacy makes residents vulnerable to misleading health information and irrational dietary choices (25). In Cambodia’s primary care system, the lack of Khmer-language tailored patient educational materials results in prevalent public misunderstanding of evidence-based glycaemic control dietary advice, forming biased perceptions that impede long-term adherence to healthy lifestyle interventions (24). For Australian Aboriginal communities, the collapse of intergenerational traditional food knowledge driven by colonisation induces cultural belief biases, creating value conflicts between mainstream low-carb diabetic guidelines and local community food customs and lowering patient engagement with metabolic remission dietary protocols (23). Collectively, knowledge deficits and culturally rooted perceptual biases weaken the efficacy of routine nutritional counselling and hinder patient compliance with standard diabetes self-management guidelines (23–25).

Notably, these attitudinal and belief biases arise from distinct contextual root causes across the two setting categories. In LMICs, such cognitive barriers are mainly attributed to nationwide systemic shortages of culturally appropriate, local-language health education resources and inherent conflicts with indigenous staple food cultures. By contrast, health belief biases among Indigenous vulnerable populations in high-income nations are primarily shaped by colonial erosion of intergenerational food heritage, compounded by domestic racial inequities in healthcare resource allocation.

##### 3.4.1.2 Knowledge and skill deficits (barrier)

Deficiencies in practical nutritional literacy and inadequate food preparation proficiency represent prominent individual-level barriers to sustained adherence to diabetic dietary regimens (19, 76). Stakeholder interviews conducted among American Indian and Alaska Native adults living with type 2 diabetes identified pervasive practical skill gaps, including impaired ability to evaluate standard food portion sizes, insufficient culinary skills for preparing fresh fruit and vegetable dishes, and the absence of time-saving meal planning strategies. Many participants declined to purchase fresh produce over concerns of spoilage caused by limited cooking proficiency, which contributed to substantial food waste and led to heavy reliance on ultra-processed convenience food options (33). In rural villages of South Africa, informal home-based caregivers exhibited profound deficits in diabetes-related knowledge before receiving standardized formal training. Most caregivers were incapable of delivering precise dietary counselling, recognizing early hypoglycaemic manifestations, supporting insulin administration for patients, or providing individualized guidance on staple food balance and portion control (90). Taken together, fundamental deficits in diabetes nutrition knowledge hinder both patients and community informal caregivers from translating professional dietary guidelines into feasible daily meal plans, which markedly undermines long-term compliance with glycaemic management protocols (33, 90).

These knowledge and skill deficits originate from context-specific drivers: rural LMIC communities suffer from national-level underinvestment in grassroots caregiver training and public nutrition education systems; meanwhile, Indigenous populations in high-income countries experience skill gaps shaped by domestic racial segregation, unequal community service provision, and racial barriers to stable access to fresh food supplies.

##### 3.4.1.3 Belief transformation promotion (facilitator)

Structured peer support programmes and context-adapted printed health educational materials function as two core enablers for positive belief restructuring among rural low-resource patients with type 2 diabetes (24, 118). Standard Khmer vernacular booklets designed for primary care facilities systematically deliver dietary and metabolic knowledge to correct fragmented, misleading lay health perceptions, gradually rectifying patients’ misconceptions of glycaemic management and fostering proactive attitudes toward long-term self-management (24). Meanwhile, organized peer support groups act as effective catalysts to reverse negative perceptions of diabetes among rural Chinese populations (118). Frequent group discussions, shared disease experiences and reciprocal emotional reinforcement strengthen participants’ sense of identity and belonging, alleviate anxiety and depression triggered by chronic illness, and replace pessimistic, passive mindsets toward treatment and dietary regulation (118). In contrast to conventional one-way nurse-led didactic education, interactive peer communication facilitates the reconstruction of rational cognition regarding diabetes self-management, enables patients to perceive tangible benefits from persistent diet and exercise interventions, and converts negative beliefs into active, consistent self-care behaviours (118). When combined with culturally tailored clinical educational toolkits, peer support frameworks work synergistically to resolve health misconceptions, elevate patient health self-efficacy, and accomplish the transition from biased negative perceptions to evidence-based positive health beliefs (24, 118).

Distinct pathways facilitate positive belief transformation across two types of resource-limited contexts: LMICs primarily rely on localized printed primary health education materials to fill nationwide gaps in public nutrition outreach and correct widespread population-level dietary misconceptions; by contrast, disadvantaged rural subgroups within middle- and high-income countries gain cognitive empowerment mostly through systematic, large-scale peer support communities that mitigate negative health mindsets shaped by socioeconomic and racial disparities.

#### 3.4.2 Outer setting

Six subthemes were identified within the Outer Setting domain to characterize external contextual determinants, covering food environments, sociocultural norms, policy and resource allocation, as well as multi-tiered social support systems.

##### 3.4.2.1 Food environment barriers (barrier)

In rural low-income communities of eastern Uganda, poverty and large household sizes constitute prominent food environment barriers for adults living with type 2 diabetes (57). Local subsistence farmers mainly rely on home-grown sweet potatoes as staple food, while animal products such as chicken, fish and eggs are financially unaffordable for most big families (43). Deep-rooted cultural perceptions shape dietary resistance: sugary and fried foods are viewed as markers of a satisfying life, and cutting these palatable items is widely regarded as “sacrificing a good life,” generating persistent psychological barriers to low-glycaemic dietary adjustments (57). Distinct food-related misconceptions exist between rural and peri-urban residents: rural participants mistakenly classify sweet potatoes as high-risk diabetic food, while peri-urban people believe only boiled meat contains low fat; few community members grasp the logic of multi-group balanced diet matching (57). Although wild fruits and vegetables grow locally, most rural households cannot produce a full range of nutrient-rich foods to meet balanced dietary standards, and peri-urban residents depend entirely on purchased food supplies, further limiting long-term access to diversified healthy meals (57).

By contrast, Indigenous diabetic residents living on rural US reservations (high-income context) are confronted with two tiers of food environment barriers. Severe food deserts constitute the primary structural constraint for American Indian tribal communities. Full-service grocery supermarkets are located roughly 90 miles away from residential areas; local convenience stores only stock overpriced, low-quality fresh produce. Limited access to private vehicles and a complete absence of public transit render routine trips for healthy groceries impractical. Most tribal households rely heavily on shelf-stable, high-sodium commodity foods provided through federal relief programmes including FDPIR and SNAP. Fixed monthly benefit allocations combined with the short shelf life of fresh produce drive families to opt for ultra-processed food items instead of low-sugar whole grains to prevent spoilage (28). Furthermore, a large proportion of older adults and low-income rural tribal residents lack smartphones and stable internet access, alongside insufficient basic digital literacy. Mobile applications designed for dietary tracking cannot be utilised consistently, forcing patients to rely on cumbersome paper recording systems and adding extra self-management burden that undermines long-term adherence (29). Taken together, geographic remoteness, insufficient local fresh food supply, absent public transportation, restrictive federal food subsidy policies and pervasive rural digital disparities jointly create multi-dimensional food environment barriers that impede consistent adherence to evidence-based diabetic dietary regimens (28, 29).

Distinct root causes drive food environment barriers across the two tiers of resource-limited settings: LMIC rural communities are mainly constrained by household poverty, large family expenditure burdens and culturally biased food perceptions, while Indigenous vulnerable populations in high-income nations face compound structural disadvantages including remote food desert geography, insufficient public transit, restrictive federal nutrition assistance rules and pervasive rural digital inequities.

##### 3.4.2.2 Sociocultural norms conflicts (barrier)

Mismatches between universal mainstream dietary guidelines and indigenous sociocultural food traditions constitute persistent sociocultural barriers that undermine diabetic dietary adherence among low-income, Indigenous and African patient cohorts (24, 27, 33). Within Cambodia’s primary healthcare system, Westernized standardized dietary guidance embedded in national chronic disease management toolkits fails to accommodate local culinary customs. Generic low-sugar and low-carb recommendations overlook carbohydrate-dominant staple foods that form the foundation of local traditional diets, which generates normative dissonance and reduces patients’ willingness to comply with clinical dietary advice (24). Among patients with type 2 diabetes in Northern Ethiopia, ingrained cultural eating patterns—including large-scale communal shared meals, routine high-starch staple consumption, and blurred boundaries between staple and indulgent foods—stand in direct opposition to portion control, calorie restriction and balanced meal frameworks advocated in clinical nutrition counselling. Even participants who receive formal nutritional education struggle to separate lifelong culturally embedded eating behaviours from evidence-based diabetic dietary regulations, resulting in unsatisfactory overall dietary compliance (27).

By contrast, Indigenous subgroups in high-income nations face distinct sociocultural conflicts shaped by colonial history. For rural American Indian reservation communities, colonial policies have caused long-term erosion of Indigenous traditional food systems, leading to profound sociocultural value conflicts. Modern evidence-based diabetic dietary guidelines that prioritize minimally processed fresh produce conflict with community cultural identity rooted in ancestral hunting, gathering and subsistence food sources. Meanwhile, federal commodity food programmes distribute shelf-stable processed foods, which violate both ancestral dietary conventions and clinical glycaemic control targets (33).

Collectively, inconsistencies between clinical dietary frameworks, community-specific food values, ancestral dietary practices and long-standing communal dining norms create pervasive normative friction. Such tension weakens the efficacy of universal lifestyle education programmes and acts as a core sociocultural barrier to sustained long-term glycaemic self-management (24, 27, 33).

Distinct sociocultural barrier drivers differentiate the two categories of resource-limited settings: LMIC populations mainly experience conflicts between imported Western diabetes dietary standards and local staple-based traditional eating customs, communal collective dining culture creates persistent barriers to portion moderation. Indigenous vulnerable groups within high-income countries suffer layered cultural contradictions stemming from colonial destruction of ancestral food heritage, alongside federal food aid systems that conflict with both ethnic food identity and clinical glycaemic targets.

##### 3.4.2.3 Policy and resource shortages (barrier)

Deficient chronic disease governance frameworks and multi-layered systemic resource shortages constitute core structural barriers that hinder standardized long-term diabetic dietary self-management across two tiers of resource-limited populations (11, 19, 38). For rural diabetic cohorts in Middle Eastern and African LMICs, incomplete national non-communicable disease policy systems and insufficient sustained fiscal health investment create persistent resource deficits: limited government healthcare budgets lead to shortages of diagnostic supplies, low-cost hypoglycaemic medications, and culturally tailored multilingual nutritional education materials; meanwhile, uneven spatial distribution of medical staff widens urban–rural diabetes care inequities, and national-level nutrition subsidy programmes for vulnerable diabetic households are largely absent (11). Household out-of-pocket medical expenditure burdens are further amplified in LMIC primary care systems such as Bangladesh, where underinvested mobile health infrastructure cannot deliver regular remote dietary reminders to low-income patients, and governmental financial support for nutrient-dense fresh produce is inadequate, making balanced glycaemic-friendly meals unaffordable for most impoverished diabetic families (38).

By contrast, rural diabetes outreach programmes targeting ethnic minority subgroups within high-income nations face distinct local fiscal constraints despite complete national medical policy systems. Evidence from rural Texas, USA, shows that practical hands-on diabetes cooking and nutrition education interventions can effectively improve patients’ dietary compliance, yet tight county-level fiscal budgets restrict instructor training frequency, limit programme population coverage, and cut free provision of multilingual low-sugar recipe leaflets and educational handouts for Hispanic, Indigenous and low-income rural residents (19).

Overall, fragmented national chronic disease policy governance, unequal spatial allocation of clinical and nutrition education resources, and the absence of targeted food welfare subsidy mechanisms jointly form multi-dimensional structural barriers that obstruct equitable, sustained dietary intervention delivery for people living with type 2 diabetes (11, 19, 38).

Distinct policy-resource constraint models differentiate the two categories of resource-limited settings: LMICs suffer fundamental nationwide systemic defects including incomplete national NCD policy architecture, insufficient central fiscal health input and universal mHealth infrastructure underinvestment; disadvantaged ethnic and rural subgroups in high-income countries operate under sound national healthcare policies but are primarily restricted by tight local county budgets, insufficient dedicated minority nutrition outreach funding and limited free culturally-adapted patient education supplies.

##### 3.4.2.4 Sociocultural facilitators (facilitator)

Embedded voluntary and community social enterprise (VCSE) institutions, participatory group learning models, and culture-based peer support constitute core sociocultural enablers that advance diabetic dietary self-management among marginalized ethnic, low-income and rural populations (33, 54, 91). In multi-ethnic disadvantaged communities across England, reputable local VCSE bodies deliver hyper-localized, culturally responsive diabetes support services, including native-language nutrition workshops, culturally matched recipe exchange and long-term peer gatherings. Such community-rooted platforms cultivate a collective sense of belonging, fill the service gap between patients and standardized NHS health education, and offer ongoing informal counselling to relieve psychological distress post-diagnosis, allowing participants to gradually modify traditional eating patterns to meet glycaemic management goals (54). In rural Bangladesh, participatory learning and action (PLA) community groups exert prominent sociocultural supportive effects by establishing gender-neutral discussion groups, holding village-wide assemblies, and cooperating with local religious leaders such as imams to embed diabetes lifestyle guidance into daily cultural communication. Community-run shared vegetable gardens and micro mutual-aid funds mitigate financial barriers to fresh food access, while collective exercise norms eliminate the social stigma restricting women’s outdoor activities, thereby synergistically lifting adherence to healthy dietary regimens (91). For American Indian and Alaska Native populations, the revitalization of ancestral food culture acts as a vital supportive driver. Nutrition education centering traditional hunted, fished and farmed ingredients harmonizes tribal cultural identity with diabetic dietary requirements. Coalitions composed of tribal elders, nutrition educators and indigenous leaders jointly develop contextually appropriate dietary guidance that respects age-old food traditions, rendering low-glycaemic balanced meals more culturally acceptable and sustainable for local residents (33). Taken together, community participatory mechanisms, ethnicity-specific peer networks, partnerships with religious stakeholders and revitalized indigenous food culture form interrelated sociocultural enablers, which mitigate conflicts over cultural eating norms and consolidate long-term compliance with evidence-based diabetic dietary plans (33, 54, 91).

Distinct sociocultural enabling mechanisms separate the two tiers of resource-limited populations: LMIC rural communities mainly rely on village-level participatory collective activities, religious stakeholder collaboration and community mutual-aid systems to simultaneously ease economic and cultural dietary barriers; disadvantaged ethnic subgroups within high-income nations gain supportive effects via formal local VCSE social organizations and systematic indigenous ancestral food cultural revitalization programmes to reconcile mainstream diabetes guidelines with ethnic food identity.

##### 3.4.2.5 Insufficient social support (barrier)

Deficient multi-dimensional social support constitutes a core psychosocial barrier that undermines sustained diabetic dietary adherence, with distinct structural gaps observed among ethnic minority groups in high-income nations and rural diabetic populations in low- and middle-income countries (18, 19, 113).

For rural Hispanic and American Indian cohorts participating in Texas community diabetes education programmes, fragmented household dietary support and underdeveloped peer communication networks severely weaken long-term healthy eating motivation (19). Most families prepare separate regular meals rather than unified low-sugar, low-fat dishes tailored for diabetic relatives, making it difficult for patients to maintain consistent dietary standards at home. Meanwhile, there is a lack of stable platforms for patients to exchange practical cooking skills and mutually supervise portion intake; without regular peer interaction, individuals easily lose self-discipline over daily diet. American Indian and Alaska Native tribal communities face dual social support deficits (33). The collapse of ancestral food-sharing communal systems caused by colonial history breaks traditional mutual aid mechanisms for healthy food access. Although federal food relief programmes exist, there is no sustained peer buddy framework for diabetic residents; patients lack long-term companions to share disease distress and adjust eating habits together, and family members rarely receive systematic dietary supervision training, resulting in insufficient daily reminders and emotional encouragement. Many participants revert to high-calorie traditional staple diets due to isolated self-management without surrounding social support.

Rural Chinese diabetic populations Rural type 2 diabetes patients in China suffer three layers of inadequate social support: insufficient tangible domestic assistance, scarce long-term emotional companionship, and low utilisation of community public health resources (113). Cross-sectional survey data show that patients with low overall social support exhibit far poorer compliance with regular meal timing, carbohydrate counting and holiday dietary restrictions. Limited family reminders and persistent negative emotional isolation easily trigger depressive moods, which further disrupt long-term glycaemic dietary management. Three dimensions of social support are all deficient in rural cohorts: low objective material support, weak subjective emotional communication with family members, and extremely low utilisation of community nutrition lectures and follow-up services. Patients with high self-reported social support score significantly higher on dietary self-management indicators such as carbohydrate estimation and food exchange application, while those lacking family and peer backing rarely master standardised diabetic meal planning methods (113).

Northern Chinese remote urban and rural patients in remote northern Chinese cities generally receive irregular, fragmented social support from medical staff, relatives and media (32). Only a small proportion of diabetics acquire systematic nutrition guidance from clinicians; most obtain scattered diet knowledge from informal channels such as wardmates and television, without sustained one-on-one family follow-up support. A large share of family caregivers cannot accompany patients to attend regular nutrition education, and there is a lack of household joint learning sessions, so families fail to form unified dietary supervision norms. Without monthly telephone follow-up and group communication mechanisms, patients cannot obtain timely targeted support when encountering dietary confusion, leading to poor long-term maintenance of balanced eating patterns (32).

Taken together, divergent social support deficits emerge across two tiers of resource-limited populations: Indigenous and ethnic minority subgroups within high-income countries are primarily hindered by collapsed traditional communal food support systems and absent structured peer buddy groups; rural diabetic residents in LMICs face triple barriers including inadequate household material and emotional support, as well as underutilised community group education and long-term follow-up support services. The universal lack of consistent family reminders, mutual peer supervision and accessible professional auxiliary support reduces patients’ nutritional self-efficacy and generates persistent confusion in long-term dietary adjustment, forming a multi-layered psychosocial obstacle to standard diabetic dietary self-management (18, 19, 32, 113).

##### 3.4.2.6 Facilitative social support (facilitator)

Multi-layered facilitative social resources including professional clinical nutrition guidance, structured community group education and sustained family engagement constitute core enablers that optimize diabetic dietary self-management and foster positive health beliefs among resource-limited populations (25, 32, 86).

In peri-urban Kenyan LMIC settings, culturally tailored diabetes education groups led by community health workers deliver continuous informational and motivational social support. The integrated intervention combining five-day offline interactive training and four-week mobile text follow-up significantly elevates residents’ diabetes risk perception, awareness of dietary intervention benefits and nutritional self-efficacy; it also eliminates psychological resistance to lifestyle modification and reduces consumption of refined grains and cooking oil while boosting fruit and vegetable intake (25).

By contrast, standardized hospital-based nutrition education serves as formal supportive infrastructure for type 2 diabetic patients in remote northern Chinese cities. Systematic nutrition lectures, individualized meal planning consultation and monthly telephone follow-ups expand patients’ professional dietary knowledge. Integrating family members into nutrition counselling provides daily behavioural supervision and long-term emotional comfort. This combined professional-family support model markedly improves patients’ food exchange and balanced matching skills, yielding statistically significant reductions in fasting and postprandial glucose concentrations (32).

Furthermore, family-centred emotional communication and spontaneous peer knowledge exchange deliver unique culturally congruent facilitative support for remote Inuit Indigenous communities on Canada’s Baffin Island (86). Most Inuit participants identify close family members as their primary long-term source of psychological and practical support; regular dialogue and mutual oversight of traditional food intake effectively mitigate diabetes-related stigma and ease negative emotions linked to dietary restrictions. Many patients voluntarily share glycaemic management experience and indigenous country food cooking skills with younger community members through oral storytelling, forming informal peer mutual aid networks to raise collective nutritional awareness. All interviewees expressed strong demand for permanent culture-specific diabetes peer groups to resolve language barriers in mainstream English educational materials and sustain consistent healthy eating motivation (86).

Overall, three complementary forms of social support — professional clinical nutrition services, structured community education platforms and long-term family emotional supervision — jointly strengthen patients’ confidence in adhering to evidence-based dietary regimens and relieve psychological distress triggered by chronic diabetic conditions (25, 32, 86).

Distinct social support development patterns emerge across two tiers of resource-limited populations: LMICs rely on community health worker-led group training and low-cost mHealth reminders to fill gaps in hospital nutrition services; middle-income countries build systematic hospital-centred nutrition education paired with family participation mechanisms; while Indigenous minorities within high-income nations depend on family bonds and culture-adapted oral peer sharing to address linguistic and cross-cultural health communication barriers.

#### 3.4.3 Inner setting

Three subthemes are summarized to reflect organizational structural, staffing and cultural factors embedded within local healthcare systems.

##### 3.4.3.1 Resource and structural inadequacies (barrier)

Widespread systemic resource shortages and structural defects constitute institutional barriers that hinder consistent diabetic dietary self-management among remote Indigenous populations and primary care patients in low- and middle-income countries (LMICs) (23, 25, 82).

For remote Ngarrindjeri Aboriginal communities in rural South Australia, geographic isolation creates persistent barriers to routine in-person nutritional follow-up (9). Local primary health clinics lack dedicated continuous glucose and ketone monitoring equipment, and culturally tailored printed dietary guidance materials are not routinely supplied within standard public health workflows. Formal recurring group nutrition workshops and long-term health coaching services are not embedded into regular care delivery systems. In addition, remote geographic location coupled with limited local retail options restricts stable access to affordable fresh produce, forming tangible obstacles to sustained low-carb dietary compliance (23).

At a cross-national systemic level, primary healthcare systems across Sub-Saharan African LMICs face universal structural and resource constraints (25, 82). In peri-urban Kenyan communities, national chronic disease governance suffers insufficient fiscal investment for standardized nutrition outreach; most grassroots clinics operate with understaffed medical teams lacking specialized nutritional training, while outdated, non-localized diabetes education pamphlets remain the only available patient resources (23). Primary care facilities in northern Ghana reflect overlapping structural deficits that disrupt consistent dietary adherence (82). Severe workforce bottlenecks are prominent: facilities have insufficient general clinicians, almost no full-time dietitians, and inadequate systematic nutrition training for frontline medical staff, alongside overly compressed consultation times that rule out in-depth dietary counselling. Clinically, stockouts of blood glucose meters and expensive test strips impede regular glycaemic self-monitoring. From a food access perspective, erratic seasonal fresh food supply and pervasive household financial hardship limit access to nutrient-dense ingredients; long travel distances to medical facilities further break the continuity of nutritional follow-up visits (82).

Collectively, geographic segregation of remote Indigenous settlements, inadequate clinical testing infrastructure, underdeveloped nutrition workforce systems, outdated non-culturally adapted educational materials, unstable fresh food supply chains and insufficient primary care fiscal investment combine to form multi-layered structural barriers preventing long-term adherence to evidence-based diabetic dietary regimens (23, 25, 82).

Distinct root causes separate resource deficits across two tiers of disadvantaged groups: Remote Indigenous communities within high-income nations mainly face geography-driven service isolation and missing culture-specific health resources under complete national medical policy frameworks; by contrast, LMIC primary systems suffer comprehensive bottom-up systemic shortages including insufficient state health budgets, nationwide nutrition workforce gaps, unintegrated local food supply chains and underdeveloped grassroots chronic disease service mechanisms.

##### 3.4.3.2 Organizational support facilitators (facilitator)

Standardized institutional organizational services act as critical facilitators of diabetic dietary self-management across diverse resource-limited populations (19, 42, 82). In rural multi-ethnic Texas of high-income America, county Cooperative Extension System launches systematic four-week bilingual cooking education programmes with instructor training, free low-sugar recipe handouts and virtual learning channels to boost participants’ healthy cooking adherence (19). Urban Malawian public hospitals set up nurse-led long-term diabetes peer groups and deliver routine dietary counselling during outpatient visits, forming hospital-based supportive organizational mechanisms (42). In Ghana’s tertiary care facilities, regular diabetes clinics provide targeted dietary guidance, yet the lack of full-time dietitians restricts the depth of institutional nutrition support (82).

Distinct institutional support gaps and delivery models separate the three contexts: High-income US counties rely on well-funded, culturally bilingual public extension systems with complete offline-online training infrastructure; urban Malawian hospitals build low-cost peer group mechanisms as a supplementary organizational tool amid limited nutrition staffing; Ghana’s tertiary clinics possess specialized diabetes consultation windows but suffer a fundamental shortage of professional dietetic manpower that limits in-depth personalized dietary intervention.

##### 3.4.3.3 Organizational and cultural barriers (barrier)

Remote island and cross-border rural medical facilities face intertwined organizational structural defects and cultural obstacles that hinder consistent diabetic dietary self-management support (74, 84).

(1) Organizational structural barriers

From an organizational perspective, isolated rural Hawaiian island clinics suffer severe shortages of specialized diabetes physicians, certified dietitians and formal self-management educators, leading to inadequate targeted chronic disease nutrition services and restricted access to telemedicine specialty care for disadvantaged Indigenous populations (74). In rural medical systems along the US Texas-Mexico border, institutional operation pressures are prominent: full-time nurse case managers are in short supply, with each practitioner assigned an unmanageably large patient caseload. Insufficient institutional funding also cuts down long-term regular follow-up and individualized one-on-one dietary counselling for low-income Hispanic diabetic patients (84).

(2) Cultural barriers

Deep-seated ethnic dietary customs exacerbate the adverse impacts of organizational resource shortages. Rural Native Hawaiian and Filipino residents retain high-fat traditional staple food cultures, while Mexican-American border families value collective household dining traditions. A large number of patients reject standardized Western dietary guidelines for diabetes, as they consider clinical nutrition advice incompatible with inherited ethnic eating norms, which greatly weakens the actual effect of hospital-led dietary intervention programmes (74, 84).

Overall, understaffed clinical specialists, overloaded case management workloads and imperfect remote medical infrastructure form core organizational bottlenecks; ingrained Indigenous and Hispanic communal food habits conflict with evidence-based diabetic dietary requirements and constitute typical cultural barriers. The two categories of limitations interact and jointly impair patients’ long-term adherence to scientific glycaemic control diets.

#### 3.4.4 Intervention characteristics

Four subthemes are extracted to interpret core attributes of intervention design, covering contextual adaptability, evidence base, cultural customization and implementation complexity.

##### 3.4.4.1 Poor adaptability (barrier)

Generic one-size-fits-all diabetes self-management interventions and educational materials suffer poor contextual and cultural adaptability, forming prominent implementation barriers that differ distinctly between high-income (HIC) and low- and middle-income (LMIC) populations.

In rural multi-ethnic counties of the United States, standard monolingual English diabetes dietary resources fail to accommodate Hispanic and Indigenous residents’ long-standing ethnic food traditions (19). Conventional universal meal guidance centres on mainstream white dietary patterns, ignoring staple foods such as corn tortillas and legumes that form the core of Latinx daily diets. Generic leaflets lack region-specific, culturally modified low-carb recipes tailored to local cooking customs; language barriers further hinder comprehension among low-literacy minority groups. Without bilingual teaching tools and ethnic-adapted meal plans, standard Western nutrition advice conflicts with community eating norms, creating persistent implementation resistance. The mismatch between mainstream intervention frameworks and minority cultural diets constitutes a critical adaptability barrier, which the bilingual CWWD cooking programme was designed to mitigate (19).

Two typical forms of poor intervention adaptability are observed across LMIC settings. First, internationally standardised chronic disease toolkits translated for Cambodia only contain general lifestyle tips without locally customised dietary guidance (24). The Khmer-language educational leaflets adopt Western nutritional logic, lacking low-carb recipes built around domestic staple crops and ignoring local communal dining habits, making universal diabetes protocols hard to execute at grassroots health centres. Second, SMS-based remote diabetes interventions in urban Bangladesh exhibit severe contextual incompatibility (55). Standardised Bengali text messages deliver unified glycaemia advice that disregards diverse household income levels, indigenous eating customs and religious taboos. Widespread shared mobile devices, unstable telecommunication infrastructure and low population literacy further reduce the usability of this digital intervention tool, as the generic message template cannot deliver personalised dietary advice fitting local daily realities.

Collectively, poor adaptability presents divergent manifestations across resource tiers: HIC disadvantaged minorities mainly face linguistic and ethnic food mismatches in mainstream monolingual intervention materials, while LMIC populations confront dual barriers of Western standard tools decoupled from local staple food systems and digital mHealth interventions unfit for regional infrastructure and population literacy levels.

##### 3.4.4.2 Evidence-based strength (facilitator)

Evidence-based nutritional and educational interventions constitute core facilitators for diabetic dietary management, with solid empirical and theoretical support in both high-income Indigenous settings and low- and middle-income African regions.

Low-carbohydrate dietary therapy is a rigorously evidence-backed core intervention to achieve type 2 diabetes remission for Australian Ngarrindjeri Aboriginal communities (23). Systematic reviews and formal consensus statements issued by the Australian Diabetes Society confirm that carbohydrate restriction effectively reduces HbA1c, ameliorates insulin resistance and optimises blood lipid indicators, enabling drug-free glycaemic remission for eligible patients (9). This dietary framework is validated by large volumes of international randomised controlled trials and mature behaviour change theories, and has been officially integrated into clinical guidelines for Indigenous community chronic disease care. Besides universal clinical evidence, the low-carb scheme was co-developed with local Aboriginal residents through participatory workshops, which enhanced cultural compatibility and greatly improved on-the-ground implementation feasibility within remote Indigenous settlements (23).

Culture-adapted diabetes nutrition education programmes also carry strong theoretical and empirical foundations for African populations (25). Built upon the Health Belief Model and global standard diabetes management guidelines, this intervention obtains reliable evidence from cluster randomised trials among peri-urban Kenyan participants. Trial outcomes demonstrate that the curriculum significantly raises residents’ diabetes knowledge and dietary self-efficacy, while cutting consumption of refined cereals and unhealthy cooking fats. The solid theoretical basis and measurable behavioural improvements make it a feasible, evidence-supported self-management tool for LMIC urban fringe diabetic groups (25).

To sum up, evidence-based facilitators present two distinct forms across resource contexts: HIC Indigenous areas adopt low-carb dietary protocols validated by national diabetes associations and co-designed with local ethnic groups; LMIC peri-urban communities rely on theory-driven, culturally tailored nutrition education interventions supported by local cluster trial data, both of which deliver proven positive effects on patients’ glycaemic and dietary self-management outcomes.

##### 3.4.4.3 Adaptability promotion (facilitator)

Customised, contextually adjusted intervention frameworks act as key facilitators by eliminating adaptability barriers and improving the cultural and personal suitability of diabetic dietary self-management, with representative practical models identified in both high-income Indigenous communities and low-income regions (23, 31, 38).

Participatory co-designed, culture-centred low-carb dietary interventions effectively boost cultural adaptability for Ngarrindjeri Aboriginal populations (23). The scheme is developed via collaborative workshops with local Aboriginal elders, fully incorporating Indigenous traditional food knowledge into intervention design. Practitioners provide community-specific meal guides, exclusive culturally compatible recipe handouts and weekly fresh ingredient supplies; meanwhile, it combines local traditional informal yarning communication modes with standardised diabetes monitoring tools. The fully culture-aligned service design greatly raises residents’ acceptance and long-term sustainability of dietary self-management behaviours within remote Aboriginal settlements.

Transtheoretical model-based staged personalised counselling (31). Clinicians first assess patients’ readiness for dietary behaviour transformation, then deliver tiered consultation and targeted problem-solving guidance matched to different behaviour change phases. This person-centred matching strategy reduces patients’ perceived dietary barriers and elevates nutrition self-efficacy, significantly improving sustained dietary adherence among low-income vulnerable populations. Localised voice mHealth reminder system for low-literacy rural Bangladeshi patients (38). Instead of written SMS content, the platform sends personalised health prompts through native Bengali audio calls to overcome text literacy limitations. All reminder information is customised to individual medication plans, dietary rules and daily schedules, with a 24-h free medical hotline available to resolve real-life lifestyle difficulties, which substantially enhances the operability and long-term compliance of diabetes self-management guidance in resource-scarce rural Bangladesh.

In summary, adaptive promotion facilitators differ across resource tiers: HIC Indigenous settings rely on participatory co-design to build culture-matched dietary programmes that respect native food customs and communication habits; LMIC regions adopt two complementary paths—stage-matched personalised behavioural interventions for general low-income groups and audio-based digital reminders tailored to low-literacy populations, both of which resolve context mismatch barriers and stabilise long-term dietary adherence.

##### 3.4.4.4 Complexity-related barriers (barrier)

Complicated multi-faceted intervention demands and fragmented, contradictory clinical guidance create widespread complexity barriers disrupting sustained diabetic dietary self-management, with distinct burdens observed in both high-income (HIC) and low- and middle-income (LMIC) populations (40, 43, 70, 94).

Food prescription (Food Rx) integrated chronic care programmes introduce layered logistical and self-care complexity for low-income diabetic patients in Florida (94). Participants must complete multiple coordinated tasks concurrently: regular clinic visits to obtain food vouchers, repeated trips to limited-hours on-site or mobile food pantries, quarterly biometric testing (HbA1c, blood pressure, BMI), and serial food security surveys at 3-, 6- and 12-month intervals. Conflicting work schedules, limited transportation and restricted pantry opening hours form persistent practical obstacles. Longitudinal repeated assessments and stacked administrative obligations gradually reduce sustained programme engagement, while a notable proportion of participants withdraw due to the overwhelming multi-step procedural complexity of combined food assistance and diabetes monitoring workflows (94).

For Iranian adults with type 2 diabetes, integrated weight management interventions that require simultaneous dietary modification, routine physical activity and sustained medication adherence impose heavy cognitive and behavioural loads on patients. A cluster of intertwined challenges further amplifies implementation difficulty, including insufficient capacity to make independent dietary decisions, frequent emotional overeating triggers, and various exercise limitations such as bodily fatigue, chronic pain and inadequate public sports infrastructure. Demographic and disease-related factors further compound intervention complexity; older adults, low-literacy, rural residents and patients with long disease durations face overlapping self-care hardships, making long-term holistic lifestyle adjustment difficult to maintain (40). Among Black South diabetic populations, inconsistent and ambiguous nutritional counselling delivered by primary care providers acts as a key complexity obstacle. Frontline clinicians without systematic nutrition training offer conflicting recommendations on staple foods such as maize porridge, leaving patients confused about permitted food categories and standard portion sizes. Most participants lack access to unified food exchange tools and clear portion measurement instructions, while fragmented clinical advice fails to take local cultural traditions, household finances and daily living realities into account. Patients are thus forced to reconcile formal dietary rules with local funeral feasts and affordable indigenous ingredients, substantially weakening their capacity to sustain long-term dietary compliance (43). In conflict-affected Democratic Republic of the Congo, standard universal diabetes guidelines bring severe practical complexity to local patients. Nutritionally recommended healthy foods are costly and rarely accessible locally, creating extra financial pressure when patients attempt to prepare separate diabetic meals for household consumption. Complex multi-task self-care burdens exacerbate the difficulty of adherence: patients need to combine rigorous diet restriction, repeated long-distance hospital visits amid regional instability and persistent medication taking. Frequent food scarcity creates a vicious cycle where individuals skip prescribed medicines due to insufficient nutritional intake. Furthermore, widespread community misconceptions frame diabetes as a curable disease exclusive to wealthy groups, leading most residents to prioritise traditional healers over the complicated long-term chronic self-management regimens promoted by medical staff (70).

To summarise, complexity barriers differ by resource tier: HIC vulnerable patients primarily struggle with stacked logistical procedures and recurring serial assessments from integrated food-medical intervention schemes; LMIC populations face overlapping burdens from multi-dimensional lifestyle requirements, disjointed clinical nutrition advice, plus resource and security-driven practical constraints that multiply self-management complexity.

#### 3.4.5 Implementation process

Three subthemes captured operational barriers and enablers during intervention rollout and ongoing delivery.

##### 3.4.5.1 Training and education deficits (barrier)

Insufficient, fragmented and unsystematic training and educational resources create universal barriers to sustained diabetic dietary self-management, with distinct gaps in workforce and patient education observed in high-income and low-middle-income contexts (24, 27, 35, 85).

In non-remote Aboriginal communities of New South Wales, systemic shortages of standardized, culturally inclusive training constitute critical workforce education deficits that impede equitable diabetes dietary management (85). The mainstream primary care training framework delivers only a half-day basic diabetes module for Aboriginal Health Workers (AHWs), covering merely superficial screening and general lifestyle knowledge, with no dedicated coursework on Indigenous-specific nutritional counselling, culturally appropriate portion guidance or trauma-informed dietary intervention for Aboriginal patients (85). No state-wide recurring refresher workshops or formal credentialing pathways are provided for frontline clinic staff serving Indigenous populations; most general practitioners receive minimal targeted training on the unique BMI thresholds, traditional staple food systems and intergenerational trauma risks that drive elevated diabetes prevalence among Aboriginal adults. Clinicians lack structured education to reconcile Western dietary guidelines with local Aboriginal food customs, and few standardized bilingual teaching manuals tailored to Indigenous cultural contexts are distributed across community clinics. Furthermore, patient-facing educational resources suffer equivalent training and design gaps: routine health lectures are delivered without formal training for facilitators on Aboriginal communication norms such as yarning, and educational materials fail to integrate locally relevant food examples due to insufficient cross-cultural educator training. These persistent training deficits lead to inconsistent, superficial nutrition advice and low patient engagement with long-term self-management programmes among Aboriginal groups, whose diabetes prevalence is 2.5–4 times higher than non-Indigenous counterparts (85).

Primary care workforce training gaps are widely observed in Cambodia’s grassroots health system: frontline staff lack standardized long-term non-communicable disease training curricula covering multi-stage diabetes screening, risk evaluation and standardized referral pathways compliant with global clinical guidelines. Although Khmer-language localized diabetes toolkits have been developed to resolve operational shortages, official training handbooks and periodic refresher workshops for clinic practitioners are still unavailable, making it difficult for grassroots medical staff to fully implement complex evidence-based dietary and metabolic management protocols, resulting in uneven service quality among different primary health facilities (24). In public medical institutions of Northern Ethiopia, clinical teams receive almost no systematic professional nutrition training; routine diabetes consultations only provide scattered oral dietary advice without unified printed teaching materials on portion control, calorie calculation and structured meal planning. The fragmented one-off health education fails to teach practical skills such as food exchange and plate diet methods, with only 1% of local type 2 diabetic participants meeting healthy eating criteria across all three core dietary dimensions, and over half of respondents showing poor performance in every eating behaviour indicator (27). Meanwhile, conventional diabetes education models in urban Chinese community clinics neglect systematic family-participation training modules. General health lectures only target individual patients rather than involving household caregivers to learn unified dietary supervision skills. Without staged personalized coaching and long-term follow-up reinforcement training, single-session knowledge popularization barely drives sustained dietary behaviour improvements, especially for patients with low baseline family support, who gain minimal benefits from incomplete, non-structured educational interventions (35).

Taken together, training deficits diverge by economic tier: HIC systems suffer from inadequate Indigenous/cross-cultural specialized training, incomplete refresher mechanisms and culturally mismatched educator training; LMIC settings face triple limitations including absent standardized clinician training systems, scarce unified printed teaching aids, and a lack of long-term family-integrated personalized patient education, all of which weaken the durability of evidence-based diabetic dietary guidance (24, 27, 35, 85).

##### 3.4.5.2 Execution process facilitators (facilitator)

Diversified layered execution mechanisms including digital remote tracking, individualized paper diet aids and culturally adapted offline group services act as complementary implementation facilitators to maintain long-term diabetic diet and exercise self-management, with differentiated mature models in high-income and low- and middle-income regions (66, 95, 109).

Interactive SMS-based SMART digital monitoring platforms form mature remote execution support in high-income settings (95). The system distributes weekly personalised text intervention modules matching patients’ individual health goals, with two-way message interaction and automatic motivational feedback to consolidate healthy living habits. Standardised active follow-up procedures are built into the platform: automatic reminder messages are sent if users fail to reply within 3 h, and research staff make outbound phone calls for participants unreachable for three consecutive days to solve technical faults and relieve behavioural burnout. All text content adopts concise plain language with resource hyperlinks to reduce comprehension burden, and flexible message sending schedules adapt to participants’ daily timetables, effectively stabilising long-term participation rates and sustaining self-management motivation.

Multi-channel regular follow-up combined with custom paper diet materials is a mainstream implementable support strategy for LMIC populations (109). The intervention carries out tiered education: group lectures on glycaemic index and glycaemic load knowledge are combined with one-to-one personalised recipe design adjusted to each patient’s activity intensity and food preferences. Weekly telephone follow-ups are arranged to check diet logs, correct irrational eating behaviours and deliver targeted guidance on fruit intake, sugar limitation and portion control. Unified 3-day diet record booklets, printable GI lists and food exchange charts are provided to standardise self-monitoring and simplify daily food selection, lowering the operational threshold of persistent dietary adherence. Culture-adapted offline structured courses and routine home visits further promote sustained diet and exercise behaviour change in low-literacy LMIC communities (66). International universal diabetes guidelines are localised into visual French slide materials and simple paper tracking forms, replacing complex calorie counting with intuitive staple portion recording rules. The 14-unit offline curriculum contains role-play, on-site cooking demonstrations and collective community sports; community health workers conduct regular home visits between sessions to supervise daily diet and exercise compliance. Low-cost local recipe brochures featuring native seasonings and household exercise manuals reduce implementation barriers, and each session adds family participation guidance to drive joint supervision by household members, significantly improving long-term adherence to healthy lifestyle regimens.

Taken together, HIC mainly relies on intelligent digital remote supervision as the core execution facilitator; LMICs adopt two matching offline paper tool + community group/home visit systems. The three layered implementation models complement each other, eliminate operational obstacles and enable continuous diet and exercise self-management among diabetic patients (66, 95, 109).

##### 3.4.5.3 Training and education promotion (facilitator)

Three distinct theory-driven, targeted educational training models serve as core facilitators to boost diabetic dietary and metabolic self-management among populations in high-income (HIC) and low- and middle-income (LMIC) settings, with differentiated design logic and implementation pathways (25, 31, 59).

Stage-matched dietary training built on the transtheoretical model (TTM) represents a universal person-centred educational facilitator applicable to underserved low-income American adults with type 2 diabetes (31). Clinicians conduct differentiated targeted training and counselling according to patients’ individual readiness to modify eating behaviours: concentrated guidance is delivered for individuals in contemplation and preparation phases to resolve perceived dietary barriers, while systematic training covering carbohydrate counting, portion management and practical dietary problem-solving is provided for participants entering the action stage. Validated brief assessment tools including the Personal Diabetes Questionnaire (PDQ), Diabetes Self-Efficacy Scale (DSES) and Diabetes Knowledge Test (DKT) are deployed in routine clinical training to rapidly identify patients’ knowledge gaps and behavioural obstacles, enabling dynamic adjustment of training priorities based on individual characteristics. Skill-oriented staged training significantly elevates diabetic self-efficacy and mitigates perceived dietary difficulties to sustain stable healthy eating and favourable glycaemic outcomes. Notably, male and socioeconomically disadvantaged subgroups require repeated, intensified training sessions to raise intervention engagement, as gender disparities exist in willingness to receive long-term dietary education (31).

Community health worker-led group diabetes education grounded in the Health Belief Model functions as a low-literacy oriented training facilitator for LMIC peri-urban adults (25). The whole training framework is fully localised to fit Kenyan cultural norms and low health literacy levels, consisting of five modular offline face-to-face courses supported by supplementary weekly WhatsApp/SMS knowledge reinforcement after offline workshops. Interactive teaching strategies including vernacular oral explanation, large visual charts and hands-on food demonstration plus teach-back verification are adopted to eliminate comprehension barriers for residents with limited education. Standard two-day pre-service systematic training is provided to all community health workers before intervention delivery, equipping frontline staff with professional counselling competency covering local staple dietary adjustment, physical activity guidance and alcohol/tobacco control. Post-training data demonstrated that this culturally tailored training significantly elevated participants’ diabetes knowledge, perceived disease susceptibility and behavioural self-efficacy, while effectively reducing refined grain and edible oil intake and boosting fruit consumption to improve long-term dietary adherence (25).

Gender-differentiated targeted nutritional training focusing on calcium and vitamin D metabolism acts as a population-specific educational facilitator for Chinese women living with type 2 diabetes (59). Distinct from Western universal dietary training frameworks, this training system fully accounts for the grain-dominated, low-dairy feature of traditional Chinese diets, which leads to chronic calcium insufficiency especially among postmenopausal females. Clinicians carry out personalised nutritional training based on patients’ serum calcium and 25-hydroxyvitamin D test results, prioritising systematic education on high-calcium dairy and legume intake, alongside fine-tuned guidance on total energy, fat and carbohydrate balance to alleviate postprandial blood glucose surges. Training content clarifies gender-based metabolic discrepancies: calcium and vitamin D supplementation delivers prominent glycaemic protective effects for women, whereas male patients require separate training targeting excessive alcohol and calorie overconsumption. Standardised gender-specific nutrition training corrects imbalanced daily dietary structures, deepens patients’ understanding of the linkage between micronutrient status, bone health and glycaemic control, and stabilises long-term blood glucose management outcomes among female diabetic populations (59).

Taken together, training and educational promotion facilitators show tiered differences across economic strata: HIC settings rely on Transtheoretical Model-based staged individualised clinical training that dynamically adjusts content by behavioural change readiness; LMIC regions adopt two complementary training pathways—community-oriented culturally adapted group training rooted in the Health Belief Model for low-literacy peri-urban residents, and gender-specific bone metabolism integrated nutrition training tailored to Chinese women’s unique dietary and metabolic characteristics. All three theory-guided educational models fill patients’ knowledge gaps, lower dietary implementation barriers and strengthen sustained adherence to evidence-based diabetic eating regimens (25, 31, 59).

## 4 Discussion

Based on the Consolidated Framework for Implementation Research (CFIR), this study conducted a scoping review of the implementation evidence of evidence-based dietary strategies for type 2 diabetes mellitus (T2DM) in resource-limited settings. For the first time, a multi-level, cross-cultural, and cross-regional comprehensive evidence mapping was completed for this specific scenario. The results confirmed that the successful implementation and translation of T2DM dietary strategies cannot be achieved by overcoming a single barrier, but rely on the synergistic adaptation of the five core CFIR domains (intervention characteristics, outer setting, inner setting, individual characteristics, and implementation process). Among them, localized adaptation (including local food substitution, simplification of visual tools, and in-depth embedding of cultural norms) serves as a core theme throughout the entire process and a key hub for bridging the gap between evidence-based evidence and complex real-world scenarios.

### 4.1 Stratified comparison of implementation determinants across two types of resource-limited settings

Consistent with the stratified thematic synthesis presented in Section 3.4, this study further distinguishes divergent root causes and manifestation patterns of implementation barriers between two categories of resource-limited populations: low- and middle-income country (LMIC) communities, and marginalised Indigenous/rural subgroups within high-income countries (HICs).

Barriers and structural constraints specific to LMICs. The core bottlenecks hindering dietary intervention translation in LMICs stem from nationwide systemic underinvestment and incomplete public health governance frameworks. At the outer setting level, fragmented national NCD policies, insufficient fiscal allocation to primary care, and underdeveloped healthy food supply chains create universal structural shortages. Within inner setting domains, grassroots clinics face persistent workforce deficits: few dedicated dietitians, compressed consultation time, and lack of standardized multilingual nutrition training materials. At the individual and intervention levels, population-wide low health literacy, staple-food-centred local culinary traditions, and unaffordable tailored educational tools amplify resistance to Western standardized dietary guidelines. Most implementation obstacles in LMICs are systemic, cross-regional, and cannot be resolved via local community interventions alone, requiring top-down policy coordination and sustained national health investment.

Barriers and structural constraints specific to marginalised subgroups in HICs By contrast, resource deprivation among Indigenous, rural and low-income minorities in high-income nations originates from domestic socioeconomic inequity and historical cultural erosion, rather than nationwide health system failure. Although complete chronic disease policy frameworks exist at the national level, localized structural exclusion persists: remote food deserts, insufficient public transit, restrictive federal food subsidy rules, and racial disparities in primary care nutrition services form multi-layered outer-setting barriers. Culturally, colonial destruction of ancestral food systems creates unique value conflicts between mainstream diabetes dietary guidance and Indigenous traditional eating customs, a challenge rarely observed in LMIC contexts. Inner setting constraints are concentrated in underfunded county-level outreach programmes rather than national infrastructure gaps, while individual-level barriers are compounded by intergenerational trauma and unequal access to culturally congruent health education.

Shared enablers and divergent optimisation pathways across both contexts Despite distinct root causes, the two groups share core facilitators including peer support, culturally adapted intervention design and community participatory mechanisms. However, feasible promotion strategies differ substantially. LMICs rely on low-cost, community health worker-led group education, local-language printed pamphlets and simple voice-message mHealth tools to overcome mass literacy deficits. For disadvantaged HIC subgroups, systematic Indigenous food culture revitalisation, bilingual professional cooking courses and formal VCSE social organisation support represent more effective pathways to reconcile guideline requirements with ethnic food identity. This stratified comparison confirms that “localised adaptation” must adopt differentiated design logic according to national income level and historical cultural context, rather than adopting a unified intervention model for all resource-limited populations.

### 4.2 Key findings and comparison with existing literature

Systematic classification of barriers and facilitators across five CFIR dimensions in the present study yields observations consistent with prevailing implementation science frameworks, while extending current evidence by elaborating targeted dietary intervention regimens, verifying intervention feasibility within low- and middle-income contexts, and broadening cross-population generalizability. Mechanistic analyses corroborate a core tenet of implementation research: isolated single-component strategies cannot mitigate systemic implementation barriers, and cross-domain synergistic adaptation constitutes a prerequisite for sustained intervention uptake. Contextualized modification serves as a pivotal solution to address practical constraints in resource-limited settings. At the intervention tier, culturally appropriate food substitution protocols and simplified operational tools alleviate two prominent outer-setting impediments, namely limited access to nutrient-dense foods and mismatches between universal dietary guidelines and indigenous sociocultural norms. Individual-centered interventions fostering cultural identity enhance treatment adherence and facilitate favorable shifts in health-related beliefs, whereas institution-based localized training remediates deficits in nutritional competency among primary care clinicians. Collectively, these tiered adjustments establish a holistic synergistic framework linking intervention design, external contextual conditions, individual recipients, and healthcare delivery organizations. The thematic co-occurrence network delineates inherent interconnections among all contributing determinants, demonstrating that implementation hurdles stem from multidimensional interactions rather than discrete single-domain factors. Knowledge and skill deficits occupy a central bridging position within the entire factor system, exerting robust associations with outer-setting constraints as well as intervention optimization strategies, thereby elucidating the integrated synergistic capacity of contextualized adaptation to resolve multi-tiered implementation bottlenecks.

These findings are highly consistent with a qualitative study by Ramani-Chander et al. (122), which evaluated 19 noncommunicable disease (NCD) scale-up projects funded by the Global Alliance for Chronic Diseases (GACD) across 20 low-resource countries. Using the CFIR framework, that study identified major barriers including outer setting factors (political influences, poor intersectoral coordination), inner setting constraints (insufficient resource investment, high staff turnover), individual-level challenges (need for sustained senior leadership support), and process-related burdens (extensive time demands for stakeholder engagement). Key facilitators included trusted networks, consistent communication, and contextually responsive adaptation.

The present study further refines these insights by focusing specifically on T2DM dietary strategies. We confirmed that localized adaptation serves as a cross-cutting mechanism that simultaneously alleviates bottlenecks across multiple CFIR domains, forming a synergistic adaptation chain of “intervention–environment–individual–implementation.” This aligns closely with the “local contextualization” mechanism highlighted as central to successful scale-up in Ramani-Chander et al. (122). However, this study places more emphasis on the depth of cultural embedding of dietary interventions (such as conflicts in festival dietary norms and participation in religious venues) and constructed a thematic co-occurrence network to visualize and quantitatively analyze the association patterns and interaction strengths among factors. Compared with the scoping review by Khalid et al. on the implementation of telemedicine for hypertension/diabetes (123), this study overlaps highly with the individual characteristics and outer setting domains (technology literacy gaps, cultural/language barriers, rural accessibility issues). However, this study focuses on non-digital or low-technology dietary strategies, emphasizing the role of simple toolkits, local food substitution, and community norm promotion rather than relying on infrastructure, thus being more applicable to extremely resource-scarce scenarios with a Health Access and Quality (HAQ) index < 50. A review by Leeman et al. on public health intervention scale-up (124) highlighted that successful implementation and scale-up rely on multi-level stakeholder engagement, policy support, and contextual adaptation across health system levels. A review by Sivakumar et al. (125) on implementation science in nutritional practices noted that CFIR applications in clinical nutrition innovation remain limited, primarily in hospitals and primary care settings. Our study extends this evidence to resource-limited community and primary care contexts, further confirming the universality and dynamic interactive value of CFIR in dietary intervention translation.

A systematic review by Means et al. on the application of CFIR in LMICs further supports the findings of this study (8), indicating that most CFIR constructs are highly compatible in LMICs, but “system characteristics” (such as health system structure, political coordination) need to be added to better capture the unique barriers in LMICs. Our analysis of included studies identified similar core gaps, including limited evidence on the inner setting and implementation process in Africa, and insufficient data on rural low-income subgroups in Asia. These findings are highly consistent with the optimization suggestions by Means et al. (8)The process evaluation of the SMART2D project by van Olmen et al. (126) (covering Uganda, South Africa, and Sweden), as well as the systematic review by Ghammari et al. on T2DM management in slum residents (127), evidence on prevention in vulnerable groups by Breuing et al. (128), and qualitative research on dietary self-management in Ghana by Hushie (129), further confirm the universality of the “core contradiction chain” (outer setting → individual characteristics → intervention characteristics → outcomes) and “systemic bottleneck” (shortage of nutrition professionals in the inner setting) identified in this study.

### 4.3 Practical and policy implications

The CFIR framework provides a systematic analytical perspective for the implementation and translation of T2DM dietary strategies in resource-limited settings. Future strategies should take localized adaptation as the core focus, focus on the multi-domain synergistic adaptation of intervention-environment-individual-implementation, and break through the systemic barriers that cannot be overcome by a single barrier, specifically implemented from four dimensions:

Policy level: Strengthen policy coordination and resource investment, integrate dietary interventions into national NCD action plans, prioritize the allocation of primary care dietitians, the construction of healthy food supply chains, and full coverage of community promotion, fundamentally addressing the systemic bottlenecks of fragmented policies and insufficient resources, and providing institutional support for the implementation of localized interventions.

Capacity building level: Conduct localized Medical Nutrition Therapy (MNT) training for primary healthcare providers, establish a multidisciplinary collaboration mechanism, and empower community health workers (CHWs) with the same background to make up for the shortage of primary nutritional capacity, so that intervention programs are adapted to local service scenarios.

Intervention design level: Deepen the “localized adaptation” strategy, focus on local food substitution, family/religious cultural embedding, and simplification of visual tools, create intervention programs that are consistent with local dietary culture and economic level, increase dietary compliance to more than 70% (9), and address implementation barriers such as cultural norm conflicts and tool inapplicability.

Implementation process level: Establish a structured support system, strengthen behaviour change through long-term follow-up, bilingual education, short message service (SMS)/APP reminders, etc., and specifically address the digital divide to ensure the accessibility and sustainability of interventions in resource-scarce scenarios.

From the perspective of the core influencing mechanisms of each CFIR domain, the improvement of food accessibility in the outer setting is a basic prerequisite for the implementation of evidence-based dietary strategies. It is necessary to optimize the availability of healthy food according to the economic level and food supply characteristics of resource-limited regions, reduce the convenient access to high-sugar and high-fat foods, and at the same time take into account traditional dietary culture, so as to realize the organic integration of evidence-based dietary requirements and local dietary patterns (11, 56). At the individual characteristics level, addressing dietary cognitive misunderstandings and improving self-management capabilities are key. It is necessary to carry out targeted health education according to the cognitive characteristics of different populations (such as female patients and rural patients), supplement practical knowledge such as cooking skills and food portion control, and at the same time pay attention to the right to speak of female patients in food procurement and dietary decision-making, reducing individual-level implementation barriers (19, 20, 56).

The localized adaptation of intervention characteristics is the core support for the implementation of evidence-based dietary strategies. It is necessary to abandon the one-size-fits-all intervention model, optimize the practicality of intervention toolkits according to the regional context and dietary customs of resource-limited regions, improve evidence-based dietary guidance programs for traditional foods, ensure that intervention suggestions are both scientific and operable, and at the same time take into account the synergy with diabetes drug treatment programs to avoid intervention conflicts (15, 21, 27). During the implementation process, it is necessary to address language communication barriers, improve the efficiency of intervention information transmission through bilingual education and improved translation services, and at the same time use multiple means such as digital tools, long-term follow-up, and peer support to enhance patients’ participation motivation and behaviour persistence, and build a “healthcare provider-family-community” collaborative support network to ensure the continuous implementation of evidence-based dietary strategies (10, 18, 52, 81, 95, 97).

In addition, combined with the conclusions of the scoping review, the implementation and translation of evidence-based dietary strategies for T2DM in resource-limited settings also need to pay attention to the differences between regions with different resource endowments, avoid the “one-size-fits-all” intervention model, and formulate differentiated implementation paths according to the specific characteristics of rural and remote areas and regions with different economic levels (122). At the same time, it is necessary to attach importance to the synergistic role of family support and community empowerment, and promote the sustainability of the implementation of evidence-based dietary strategies by guiding the whole family to participate in dietary planning and strengthening the professional guidance capacity of the community, so as to promote the transformation of intervention effects from short-term clinical indicator improvement to long-term behaviour habit formation and health literacy improvement (9, 11, 26). Future research can further focus on the key bottlenecks in the implementation of evidence-based dietary strategies in resource-limited settings, explore more targeted and scalable localized intervention programs, and provide more abundant evidence-based support for clinical practice and research in this field.

### 4.4 Study limitations

As a scoping review, this study did not conduct a traditional risk of bias assessment, but only performed descriptive grading of study design types and reporting completeness, which may affect the interpretation of evidence strength; included studies were limited to Chinese and English, which may miss studies in other languages; although the operational definition of “resource-limited” was based on the World Health Organization (WHO) and Institute for Health Metrics and Evaluation (IHME) standards (HAQ index < 50), cross-study heterogeneity still exists; in addition, most included studies were cross-sectional or short-term follow-up (only 12% of long-term studies with follow-up > 2 years), resulting in relatively weak evidence on long-term sustainability.

### 4.5 Future research directions

Priority should be given to the following in future research: (1) Hybrid effectiveness-implementation trials to evaluate both clinical outcomes and implementation processes; (2) Multi-center longitudinal studies targeting specific gaps to verify the long-term cost-effectiveness of “local adaptation” strategies; (3) Hybrid interventions integrating digital tools and community empowerment to address the digital divide; (4) Building on the updated CFIR version 2.0 framework and supplementing the “system characteristics” domain [referring to the suggestions by Means et al. (8)], combined with the RE-AIM or ERIC implementation strategy classification, to design scalable intervention toolkits.

## 5 Conclusion

The long-term sustainable implementation of evidence-based dietary interventions for type 2 diabetes relies on coordinated optimization across all five domains of the CFIR framework, and localized cultural adaptation acts as the core link bridging universal clinical guidelines and real-world practice in resource-limited settings. This scoping review systematically synthesizes cross-regional and multi-layered evidence, establishing an integrated analytical framework to support the design of contextually appropriate dietary management programmes. Several limitations exist in this study: only Chinese and English literatures were retrieved, and studies with long-term follow-up occupy a small proportion of all included evidence, which restricts the reliability of conclusions on long-term intervention sustainability. For subsequent research, hybrid implementation-effect trials are recommended; researchers can combine the RE-AIM framework to develop standardized, scalable locally-adapted intervention toolkits for resource-scarce populations.

## Data Availability

The datasets analyzed in this study are publicly available in PubMed, Web of Science, CNKI (China National Knowledge Infrastructure), Wanfang Data, and VIP Chinese Journal Databases. All included studies are cited in the manuscript, and the full texts can be accessed through the respective journal platforms. No new data were generated in this study.
