# Supplementary material for: Implementation and translation of evidence-based dietary strategies for type 2 diabetes in resource-limited settings: a scoping review based on the CFIR framework
Source: Front Public Health. 2026 Aug 6;14:1870603. doi: 10.3389/fpubh.2026.1870603 (PMC13490071; doi:10.3389/fpubh.2026.1870603)
Supplement: Supplementary file 2 [file Table_2.DOCX]

Table 1

| **First-level dimension** | **Second-level theme** | **Frequency**  **(n=113)** | **Representative citations** |
| --- | --- | --- | --- |
| Individual characteristics | Attitudes and belief biases^[15–17, 22–23, 26–29, 32, 34–40, 42–46, 48, 51–52, 54, 56, 58, 60–61, 63, 66–68, 70–71, 79–80, 82–84, 89, 91–92, 94–95, 98, 105–106, 109, 113, 115–116, 118–119, 121–124, 127]^ | 60/113 | In peri-urban Kenyan communities, residents demonstrate flawed baseline health beliefs featured by low perceived susceptibility to diabetes, limited recognition of the benefits brought by dietary adjustments and poor nutrition self-efficacy; inadequate diabetes-related knowledge renders residents susceptible to misleading health information and unhealthy dietary decisions [17]. In Cambodia’s primary healthcare context, the absence of Khmer-adapted patient educational materials leads to widespread public misunderstanding of evidence-based glycaemic control dietary advice, forming biased attitudes that hinder sustained adherence to healthy lifestyle regimens [16]. For Australian Aboriginal groups, colonisation-induced breakdown of intergenerational traditional food knowledge creates cultural belief biases, generating dissonance between mainstream low-carb diabetes guidance and community food values and reducing participants’ willingness to follow metabolic remission dietary plans [15]. |
|  | Knowledge and skill deficits^[16,20–21,23–24,27–31,33,37–38,41–50,52–56,58–63,65–66,69–83,85–96,98–100,102–105,107–113,115–123,125–127]^ | 85/113 | Among American Indian and Alaska Native adults living with type 2 diabetes, multi-group stakeholder interviews revealed widespread practical skill gaps, including inadequate ability to judge standard food portion sizes, insufficient cooking skills for fresh fruits and vegetables, and a lack of time-efficient meal preparation strategies. Many participants avoided purchasing fresh produce due to worries about spoilage resulting from poor cooking know-how, which generated frequent food waste and pushed them to rely heavily on high-processed convenience foods [25]. In rural South African villages, untrained home-based carers demonstrated severe diabetes knowledge deficits prior to formal standardized training; the majority were unable to provide precise dietary counselling, identify hypoglycaemic manifestations, assist patients with insulin administration, or deliver targeted advice on balanced staple intake and portion management [82]. |
|  | Belief transformation promotion^[16,19,22,28,33,35–59,62–64,68–69,73–75,79,82–84,88,92,94–98,101,104,106–108,111,113–120,124,127]^ | 52/113 | Standard Khmer vernacular educational booklets developed for primary care clinics deliver systematic dietary and metabolic knowledge to rectify fragmented, misleading lay health perceptions, gradually reshaping patients’ biased understandings of glycaemic management and cultivating positive attitudes toward long-term self-care [16]. Meanwhile, structured peer support groups serve as powerful catalysts for transforming negative diabetes-related beliefs among rural Chinese patients [110]. Through regular group discussions, shared disease experience and mutual emotional encouragement, participants gain a stronger sense of identity and belonging, relieve anxiety and depression caused by chronic disease, and abandon pessimistic, passive attitudes toward treatment and diet control [110]. Compared with one-way routine nurse-led education, peer interactive communication helps patients rebuild rational cognition of diabetes self-management, recognize the tangible benefits of sustained diet and exercise intervention, and convert negative health beliefs into proactive, consistent self-care behaviours [110]. |
| Outer Setting | Food environment barriers^[^****^15,17,20–24,26,28,30,39,60,64–65,67,73,85,92,96^]**** | 18/113 | Multiple structural food environment barriers undermine consistent diabetic dietary adherence among rural and Indigenous populations, creating persistent obstacles to accessing balanced, glycaemia-friendly meals. For rural American Indian reservation communities, severe geographic food deserts represent a core environmental constraint: the nearest full-service supermarket can be nearly 90 miles away, while local small convenience and dollar stores only stock limited, low-quality fresh produce at inflated prices, discouraging regular purchases of fruits and vegetables critical for blood sugar control. Transportation deficits compound this barrier, as most community members lack reliable private vehicles and there is no public transit system to reach distant grocery retailers, forcing reliance on shelf-stable, high-sodium commodity foods distributed via federal food assistance programmes. Even with government food subsidies such as SNAP and FDPIR, monthly fixed allotments and short fresh produce shelf lives lead participants to prioritize ultra-processed convenience foods over whole-grain, low-sugar options to avoid food waste [20]. Compounding physical food access limitations, rural diabetic individuals confront digital food environment barriers when adopting telehealth self-management tools; large proportions of elderly and low-income rural residents lack smartphones, steady internet access and basic digital literacy to operate diet-tracking mobile software, rendering app-based food logging unworkable and forcing cumbersome paper-based dietary recording as an alternative [21]. |
|  | Sociocultural norms conflicts^[15–20,22,24–25,27,29,31–34,40,43–45,53,56,60,63–64,68–69,72–73,78–79,81,83,87,93,97,100,104,108,110–111]^ | 40/113 | In Cambodia’s primary care context, standardized Western-style diet guidance embedded in national chronic disease toolkits fails to align with local cultural eating customs; generic low-sugar, low-carb recommendations overlook staple carbohydrate-based traditional meals widely consumed by residents, creating normative dissonance that weakens patients’ willingness to adopt clinical dietary advice [16]. For rural American Indian reservation communities, long-standing disruption of Indigenous traditional food systems driven by colonial policies generates profound sociocultural norm conflict: modern evidence-based diabetic nutrition guidance prioritizing low-processed produce clashes with community cultural identities centered on historic hunted, gathered and cultivated Indigenous foods, while federal commodity food programs distribute shelf-stable processed goods that conflict with both ancestral dietary norms and glycaemic management requirements [25]. Among type 2 diabetes patients in Northern Ethiopia, deeply entrenched cultural eating routines featuring large portioned communal meals, regular high-starch staples and limited separation of healthy and indulgent food customs directly contradict structured portioning, calorie-limiting and balanced meal norms promoted in clinical nutrition counselling; even patients receiving formal nutrition education struggle to decouple lifelong sociocultural eating habits from evidence-based diabetic diet rules, resulting in overwhelmingly poor overall dietary adherence [19]. |
|  | Policy and resource shortages^[17–19,21–26,28–31,33–36,39–40,42,44,46–47,49,50–53,55–63,66,68–69,71,73–74,79–83,85–86,89–90,92,94,102,104,117,118,125,126]^ | 65/113 | In Middle Eastern and African healthcare systems, incomplete national noncommunicable disease policies fail to allocate sufficient funding for sustained nutrition education, affordable diabetic food subsidies, and widespread training of specialized diet educators; limited government healthcare expenditure creates shortages of testing supplies, low-cost anti-diabetic medications, and culturally tailored patient educational materials, while uneven distribution of medical staff widens care disparities between rural and urban populations [3]. Rural U.S. community extension programs also face persistent resource constraints: though hands-on diabetes cooking curricula can effectively improve healthy eating behaviors, limited county budgets restrict educator training frequency, reduce program scale, and limit the provision of free low-sugar, low-sodium recipe resources and multilingual handouts for ethnic participants [11]. In Bangladesh’s primary care context, weak public health financing policies result in extremely high out-of-pocket medical costs for diabetic households; insufficient state subsidies for fresh nutritious foods, combined with inadequate investment in mobile health infrastructure, leave many low-income patients unable to afford balanced meals or access regular remote lifestyle reminders that support diet adherence [30]. |
|  | Sociocultural facilitators^[3, 6, 15, 25, 26, 28,46, 51, 65, 72, 76, 78, 83,85, 108]^ | 15/113 | In multi-ethnic deprived communities in England, trusted local VCSE services deliver hyper-local, culturally tailored diabetes support including ethnic language nutrition sessions, culturally appropriate recipe sharing and long-term peer group gatherings; these community embedded structures build a sense of belonging, bridge gaps between patients and formal NHS education, and provide sustained informal guidance that mitigates emotional distress after diabetes diagnosis, enabling participants to gradually adjust cultural eating habits aligned with glycaemic targets [46]. In rural Bangladesh, participatory learning and action (PLA) community groups function as powerful sociocultural enablers by setting up gender-inclusive discussion circles, organising village collective meetings and partnering with local religious leaders such as imams to integrate diabetes lifestyle advice into daily cultural communication; community-led kitchen gardening collectives and small mutual aid funds also ease financial barriers to fresh produce, while shared group exercise norms reduce social stigma against women’s outdoor physical activity, jointly boosting compliance with healthy dietary practices [83]. For American Indian and Alaska Native communities, reviving traditional Indigenous food culture serves as a critical sociocultural facilitator; centering ancestral hunted, fished and farmed ingredients in nutrition education reconciles tribal cultural identity with diabetes dietary rules, and community stakeholder coalitions combining elders, nutrition educators and tribal leaders co-design context-fit eating guidance that respects long-standing sociocultural food customs, making balanced low-glycaemic diets more culturally acceptable and sustainable [25]. |
|  | Insufficient social support^[4, 11, 14, 27, 33, 34, 36, 41, 42, 45, 55, 58, 60, 62, 64, 67, 70, 73, 74, 85, 86, 96, 99, 102, 104, 105,110]^ | 27/113 | For rural Chinese adults with type 2 diabetes, inadequate objective family assistance, scarce subjective emotional comfort and low utilisation of available social resources significantly impair their compliance with dietary rules; patients with low overall social support show far poorer performance in following regular meal timing, carbohydrate counting and holiday dietary restrictions, while severe depressive emotions easily arise without family reminders and psychological comfort to sustain long-term diet control [105]. In rural multiethnic Texas communities, participants often struggle to maintain consistent diabetic meal plans due to a lack of household support: many families prepare separate regular dishes rather than unified low-sugar, low-fat meals for diabetic members, and limited peer communication channels leave patients without shared experience exchange and mutual supervision on healthy cooking and portion management [11]. |
|  | Facilitative social support^[1, 2, 3, 4, 5, 6, 8, 9, 10, 13, 18, 22, 24, 28, 33, 34, 40, 46, 50, 52, 54, 55, 57, 58, 61, 62, 65, 67, 69, 75, 78, 80, 90, 92, 93, 98, 103, 105, 106, 109]^ | 40/113 | In peri-urban Kenyan settlements, culturally adapted diabetes group courses run by community health workers deliver persistent informational and motivational social support; five consecutive days of offline interactive learning sessions plus four weeks of follow-up mobile messages significantly raise participants’ perceived diabetes susceptibility, perceived benefits of dietary modification and nutritional self-efficacy, eliminating psychological obstacles and driving reduced consumption of refined grains and cooking oil alongside higher fruit intake [17]. For adults with type 2 diabetes living in remote northern Chinese urban areas, standardized hospital-led nutritional education constitutes formal supportive resources: systematic nutrition lectures, one-on-one personalized meal planning coaching and monthly telephone follow-ups enrich patients’ dietary knowledge, while involving family members in nutrition counselling delivers stable daily supervision and emotional support. The integrated professional and familial support system greatly lifts participants’ mastery of food exchange systems and balanced meal arrangement, and yields statistically significant declines in fasting and postprandial blood glucose [24]. |
| Inner Setting | Resource and structural inadequacies^[15,17,20–21,24,26,30–32,35,41,51,55,57,69,74,77,81,83,86,89–90,97,102,104,124]^ | 26/113 | 1.Multiple structural and resource deficits undermine consistent diabetes self-management for Ngarrindjeri Aboriginal community members living in remote regional areas. Geographical isolation restricts frequent in-person nutrition support, and local primary health services lack dedicated continuous glucose and ketone monitoring tools as well as culturally adapted dietary guidance materials. Routine public health service models do not deliver ongoing group nutrition sessions and sustained health coaching, while limited access to affordable fresh produce creates practical barriers to following recommended low-carb dietary approaches [15] 2. Low- and middle-income country primary healthcare systems are constrained by structural medical shortages that impede systematic diabetes nutrition counselling, including insufficient health workforce quantity and expertise, inadequate diagnostic screening equipment, and a scarcity of standardized, up-to-date chronic disease educational resources [17]  3. Multiple structural and resource inadequacies in Ghana’s primary healthcare system and local living environments hinder consistent diabetic dietary self-management. System-level constraints include insufficient trained clinical workforce, scarce dedicated dietitians, limited formal nutrition training for general providers, and compressed consultation time that restricts detailed dietary guidance. Clinically, persistent shortages of glucometers and expensive testing strips impede routine blood glucose monitoring. In terms of educational resources, mainstream diabetes pamphlets are not culturally tailored to local staple foods, lacking practical indigenous meal guidance. Beyond medical infrastructure, seasonal erratic supply of fresh produce and widespread household financial hardship create material barriers to maintaining balanced low-carb diets, while long travel distances to hospital clinics further cut off sustained nutrition follow-up support [74] |
|  | Organizational support facilitators^[26, 30, 34, 36, 38–39, 42, 50, 57, 67, 70, 72, 74, 76, 85–86, 88–89, 91, 93, 103]^ | 21/113 | 1. Organized medical support acts as a key facilitator for elderly Bai patients’ diabetes self-management. Hospitals provide multidisciplinary professional guidance, culturally tailored health education classes and standardized step-by-step intervention procedures. Integrated medical alliance links township clinics to deliver accessible, bilingual diabetes counselling for rural seniors, while systematic physical examination reminders and hands-on operation training further strengthen patients’ long-term dietary and medication adherence[103] 2. Limited organizational enablers for diabetic dietary management exist within local tertiary hospitals. Dedicated weekly diabetes clinics provide stable institutional platforms where nurses, physicians and full-time nutrition staff deliver personalised dietary counselling to patients. Informal internal case conferences among care teams also allow providers to exchange practical nutrition guidance, supporting consistent self-care advice for type 2 diabetes patients [74].  3. Formal hospital diabetes care systems constitute core organizational facilitators for type 2 diabetes self-management. Clinical nurses and clinicians deliver continuous dietary and physical activity counselling during routine outpatient visits, and they also remind patients of healthy lifestyle rules when encountered in community public spaces. Urban public hospitals sustain structured diabetes peer support groups led by retired nurses, creating a regular institutional platform for patients to exchange practical diet and exercise self-management experience. Such hospital-based professional guidance and peer group systems form stable organizational support to improve patients’ long-term adherence to recommended healthy behaviours [34] |
|  | Organizational and cultural barriers^[21，36，66，76，102]^ | 5/113 | 1.Remote rural islands suffer severe shortage of diabetes specialists, dietitians and professional self-management educators, leading to insufficient specialized chronic disease services.[66]  2. Healthcare system dependency: reliance on MSF (Médecins Sans Frontières) support; the local health department lacks ownership and sustained investment, raising concerns about project sustainability（p7-p8）[76]  3. Misaligned treatment priorities: antihypertensive and statin medications cost less than sulfonylureas or insulin, yet glucose-lowering drugs are more commonly used in practice, while lipid management is neglected （p20）[102] |
| Intervention characteristics | Poor adaptability (Barrier)^[^****^16, 47, 49, 51, 57, 79, 90, 93, 99, 101, 121, 123, 126–127]^**** | 14/113 | 1The SMS-based diabetes intervention shows obvious poor contextual adaptability as a digital self-management tool. First, national low literacy rates create comprehension barriers for patients to grasp professional diet and glycaemic knowledge delivered via brief standardized text messages. Second, one-size-fits-all generic SMS content is not tailored to local Bangladeshi daily eating customs, religious lifestyles and diverse household economic conditions, lacking individualised lifestyle guidance. Third, uneven mobile ownership, shared household phones and unstable communication infrastructure hinder consistent message reception, making the intervention unsuitable for low-resource groups without private digital devices [47]2. Low calcium and vitamin D dietary patterns represent a critical adaptive barrier for Chinese female patients with type 2 diabetes. The traditional Chinese grain-and-vegetable-based diet lacks dairy and calcium-rich foods, making it hard for women to reach protective calcium intake levels; postmenopausal women face aggravated bone metabolism defects and insulin resistance due to long-term insufficient calcium and serum 25OHD, further weakening the response to standard diabetic diet and glycaemic control interventions. Additionally, most diabetic females reduce intake of calcium-containing animal foods after diagnosis, which creates persistent incompatibility between routine diabetes dietary guidance and their actual long-term eating habits [51]3. Primary diabetes care in Cambodia faces prominent contextual poor adaptability barriers. Standard international chronic disease management guidelines and generic lifestyle education materials lack deep localisation to fit Cambodian staple food culture and rural living contexts. Though the newly developed toolkit contains basic diet advice leaflets translated into Khmer, these universal lifestyle materials do not provide customised meal plans, region-specific low-carb recipes or culturally tailored dietary operation guidance based on local traditional ingredients. Primary health facilities only hold general diet suggestions without context-adapted counselling tools, creating incompatibility between standard diabetes dietary advice and residents’ long-term indigenous eating habits[16] |
|  | Evidence-based strength (Facilitator)^[15,21,29,32,43,49,51–52,61–62,67–68,75,77,84,93,95,100–103,105,108–110,112–113,115–116,118,121–124,126–127]^ | 36/113 | 1. Low-carbohydrate dietary intervention carries robust clinical evidence and serves as a key evidence-based enabler for Aboriginal T2D remission programmes. Multiple systematic reviews and the official clinical consensus statement from the Australian Diabetes Society confirm carbohydrate-restricted nutrition therapy delivers meaningful improvements in HbA1c, insulin resistance and lipid profiles, and even enables medication-free type 2 diabetes remission. This intervention is underpinned by abundant international RCT evidence and established behavioural change frameworks, forming a formal guideline-endorsed clinical tool for community-led Indigenous chronic disease care. Beyond universal clinical evidentiary support, the low-carb framework was refined via Aboriginal co-design workshops to boost local cultural suitability, further strengthening its implementability among Ngarrindjeri Aboriginal residents[15] 2. This diabetes nutrition intervention gains solid evidence support from the Health Belief Model and global diabetes guidelines. Cluster randomised trial data prove the culturally tailored educational programme effectively lifts participants’ diabetes awareness, nutrition self-efficacy and reduces intake of unhealthy refined grains and cooking oils, forming a theory-backed, implementable self-management tool for peri-urban African populations [17] |
|  | Adaptability promotion (Facilitator)^[15,18,21–25,30,35,41,44,49–50,53–54,58–60,63–64,67,69–70,72,78–79,81,84,86–87,90,92,95–101,103,108,111–112,116–118]^ | 46/113 | 1. Co-designed, culture-centred low-carb intervention acts as a key adaptability enabler. Developed via Aboriginal elder workshops, the programme integrates Indigenous food knowledge and creates custom meal guides, recipe resources and weekly ingredient boxes fitting local traditional food access. It combines Aboriginal yarning-style counselling with standard diabetes management tools to align intervention design with community cultural norms, greatly boosting the acceptability and sustainability of dietary self-management among Ngarrindjeri Aboriginal residents [15]  2. Stage-tailored, individualized intervention planning serves a critical adaptability facilitator. Based on the Transtheoretical Model, clinicians can assess patients’ readiness to change diet first and deliver differentiated counselling and targeted problem-solving guidance matching their behavioural stages. This person-centred matching reduces perceived dietary barriers and strengthens diabetes self-efficacy, which significantly boosts long-term dietary adherence among low-income underserved diabetic populations [23]3. Locally tailored mobile voice reminder system acts as a strong adaptability enabler for diabetic self-management. The m-health intervention delivers personalised health guidance in native Bengali language via voice calls, avoiding literacy barriers caused by text messages. All reminder contents are customized to individual patients’ medication plans, dietary needs and exercise conditions, and patients can access free 24/7 physician consultation hotlines anytime to solve real-time lifestyle difficulties. Such language-friendly, individualised remote support greatly raises the usability and long-term adherence of diabetes self-management guidance among low-literacy Bangladeshi patients [30] |
|  | Complexity-related barriers^[32,35,62,75,84,86,93,96,98]^ | 9/113 | 1. Multi-layered self-management requirements form prominent complexity barriers for Iranian type 2 diabetic patients. Weight loss intervention relies on integrated diet adjustment, regular exercise and long-term medication compliance simultaneously, which imposes heavy cognitive and behavioral burdens on patients. Multiple interrelated obstacles coexist: inadequate dietary decision-making capacity, frequent emotional eating temptations, and diverse exercise barriers including fatigue, pain and limited public sports facilities. Besides, demographic and disease variables compound intervention complexity — elderly, low-literacy, rural and long-duration diabetic patients face overlapping self-care difficulties, making integrated lifestyle modification hard to sustain [32]2. Confusing, inconsistent dietary guidance creates major complexity barriers for Black South African type 2 diabetes patients. Primary care staff lack systematic nutrition training and deliver conflicting food advice; some clinicians forbid staple maize porridge while others permit it, leaving patients uncertain about allowed foods and portion standards. Few participants receive standardized food exchange charts, and most cannot grasp accurate portion control rules without clear measuring guidance. In addition, patients receive fragmented advice without integrated cultural, economic and daily living considerations, forcing them to struggle to reconcile clinical diet rules with local traditional food customs, funeral feasts and affordable household ingredients, which greatly reduces sustained dietary adherence [35]3. Standard diabetes dietary guidance creates severe implementation complexity for conflict-affected Congolese patients. The recommended healthy foods are unaffordable and scarcely available locally, forcing patients to struggle to separate special diabetic meals from household regular diets and bear extra economic burden. Meanwhile, the multi-layered management burden compounds complexity: patients must combine strict diet control, regular long-distance clinic trips amid regional unrest and consistent medication intake; food shortages often lead them to skip medicines, forming a vicious cycle of unmanageable self-care demands. Additionally, local communities hold misinformed perceptions of diabetes as a curable rich people’s illness, and many rely on traditional healers rather than following complicated long-term chronic care regimes [62] |
| Implementation process | Training and education deficits^[16,19,27,33,36,38,48–49,51,57,60,77,89,120]^ | 14/113 | 1.Primary care staff in Cambodia face notable training shortages for non-communicable disease management. Local health centres have limited prior experience delivering standardized diabetes and hypertension services; there is no systematic, sustained training curriculum to support frontline workers in mastering multi-step screening, risk assessment and referral workflows aligned with international NCD guidelines. While the locally adapted Khmer-language toolkit was designed to fill operational gaps, formal supporting training manuals and regular refresher sessions for clinic personnel remain absent, leaving grassroots providers unable to fully and accurately execute complex evidence-based care protocols, which generates inconsistent diabetes management quality across facilities [16] |
|  | Execution process facilitators^[24, 58, 61, 87, 90, 101, 105]^ | 7/113 | 1. Multi-layered interactive SMS follow-up forms core execution enablers to sustain participant engagement. The digital SMART system delivers personalised weekly text modules tailored to users’ self-prioritised health goals, with two-way reply triggers and automated encouraging auto-replies to reinforce healthy behaviour. Proactive follow-up protocols are embedded: auto reminder messages are sent for non-response after 3 hours, and research staff conduct outbound phone calls if users fail to reply for three consecutive days to resolve technical or motivational barriers. Short, plain-language content with supplementary hyperlinks lowers comprehension burden, while flexible message delivery times match participants’ daily schedules, jointly maintaining steady response rates and self-management motivation[87]2. Multi-channel regular follow-up and individualized paper diet plans act as core implementation enablers for dietary behavior modification. The intervention adopts layered education delivery, including group lectures on GI/GL knowledge and one-on-one customized recipe design based on patients’ daily activity and food preferences. Weekly telephone follow-up is arranged to track dietary records, correct unreasonable eating habits, and provide timely targeted guidance on fruit intake, sugar limitation and food portion management. Uniform 3-day dietary record notebooks are distributed to standardize self-monitoring, while printable GI/GL food tables and food exchange lists simplify daily food selection operations, lowering the operational difficulty of long-term dietary adherence[101]3. Culturally adapted offline group intervention tools and multi-layered on-site support constitute core implementation enablers for dietary and physical activity behavior change. The program converts abstract international diabetes guidelines into pictorial French PowerPoint materials and simplified paper tracking logs tailored for low-literacy local residents, replacing complex calorie counting with intuitive staple portion recording rules. Structured 14-session offline group courses integrate role-play, food cooking demonstrations and neighborhood exercise group practice; community health workers conduct regular home visits between sessions to follow up participants’ daily diet and workout implementation. In addition, culturally matched low-cost recipe booklets for local traditional sauces and at-home workout guide handouts lower operational barriers, while family participation guidance embedded in each session mobilizes household cooking partners to jointly maintain healthy eating habits, significantly lifting long-term lifestyle adherence [58] |
|  | Training and education promotion^[17,20,21,22,23,24,25,28,29,31,32,33,34,37,38,40,41,43,44,46,49,52,53,55,56,59,62,66,69,70,87,88,89,92,93,98,99,104,115,116,120 ]^ | 40/113 | 1.Theory-driven, culturally tailored group health education delivered by trained community health workers serves a core facilitator for diabetes dietary and metabolic management. All educational sessions adopt low-literacy teaching strategies including plain local language, large-font visual charts, food demonstrations and teach-back verification to lower comprehension barriers. The intervention adopts modular curriculum covering nutrition, staple food selection and portion control; after offline face-to-face courses, weekly personalised WhatsApp/SMS text reminders are continuously delivered to consolidate dietary knowledge and reinforce healthy eating behaviours. Systematic pre-service training equips community health workers with standardized behavioural guidance skills rooted in the Health Belief Model, enabling them to deliver targeted counselling and raise participants’ disease awareness, nutrition self-efficacy and long-term dietary adherence, ultimately reducing refined grain and oil intake and optimising glycaemic indicators [17]2. Stage-tailored diabetes dietary education and targeted psychological counselling serve core facilitators to boost patients’ self-management willingness and adherence. Healthcare providers can deliver differentiated education matching individuals’ readiness to change under the transtheoretical model (TTM): for patients in contemplation and preparation stages, focused guidance shall be provided to reduce perceived dietary barriers; those entering the action stage need systematic training on carbohydrate counting, portion control and dietary problem-solving skills. Standardized brief assessment tools (PDQ, DSES, DKT) help clinicians quickly identify patients’ knowledge gaps and behavioural obstacles, so as to adjust educational focus dynamically. Enhancing diabetes self-efficacy via skill-oriented education can effectively lower perceived diet-related difficulties, promote long-term healthy eating behaviours and optimise glycaemic management outcomes. Notably, male patients and low-income underserved groups require intensified, repeated dietary education to improve their engagement with self-management plans[23]3. Targeted dietary education guided by bone metabolism and glycaemic indicators is an effective intervention measure for Chinese female type 2 diabetes patients. Current Chinese residents generally have insufficient calcium intake due to grain-based traditional eating patterns with limited dairy consumption, which elevates diabetes risk especially for postmenopausal women. Clinical nutrition education should combine blood 25-hydroxyvitamin D and serum calcium test results to deliver personalized dietary guidance: prioritize popularizing high-calcium food intake advice (dairy products, bean food) for female patients, appropriately adjust total energy and fat intake plans, and balance carbohydrate supply to reduce postprandial blood glucose spikes. Differentiated nutrition education by gender is required, as calcium and vitamin D supplementation only shows protective effects on women’s glycaemic control, while male patients need focus on limiting excessive alcohol and total energy intake. Standardized nutritional knowledge popularization can correct unbalanced daily dietary structures, raise awareness of bone-nutrition-glycaemia interactions, and support long-term blood glucose management [51] |

.
