# Supplementary material for: Implementation and translation of evidence-based dietary strategies for type 2 diabetes in resource-limited settings: a scoping review based on the CFIR framework
Source: Front Public Health. 2026 Aug 6;14:1870603. doi: 10.3389/fpubh.2026.1870603 (PMC13490071; doi:10.3389/fpubh.2026.1870603)
Supplement: Supplementary file 3 [file Table_1.DOC]

Table S1 Full search strategy of PubMed database

| **Set #** | **Core Concept** | **PubMed Search Query** |
| --- | --- | --- |
| #1 | Disease | "Diabetes Mellitus, Type 2"[Mesh] OR type 2 diabet*[tiab] OR T2DM[tiab] |
| #2 | Intervention | "Diet Therapy"[Mesh] OR "Nutrition Therapy"[Mesh] OR diet[tiab] OR nutrition[tiab] OR "medical nutrition therapy"[tiab] OR dietary intervention*[tiab] OR dietary management[tiab] |
| #3 | Implementation-related factors | implement*[tiab] OR barrier*[tiab] OR facilitator*[tiab] OR adapt*[tiab] OR "CFIR"[tiab] |
| #4 | Setting & Context | (resource-limited[tiab] OR "low-resource"[tiab] OR "developing countr"*[tiab] OR rural[tiab] OR remote[tiab] OR underserved[tiab] OR LMICs[tiab] OR "low- and middle-income countries"[tiab] OR "China"[Mesh] OR "India"[Mesh] OR "Africa"[Mesh] |

Overall retrieval formula：#1 AND #2 AND #3 AND #4
